# A model of infection in honeybee colonies with social immunity

**DOI:** 10.1371/journal.pone.0247294

**Published:** 2021-02-22

**Authors:** Teeraphan Laomettachit, Monrudee Liangruksa, Teerasit Termsaithong, Anuwat Tangthanawatsakul, Orawan Duangphakdee

**Affiliations:** 1 Bioinformatics and Systems Biology Program, School of Bioresources and Technology, King Mongkut’s University of Technology Thonburi, Bangkok, Thailand; 2 Theoretical and Computational Physics (TCP) Group, Center of Excellence in Theoretical and Computational Science Center (TaCS-CoE), King Mongkut’s University of Technology Thonburi, Bangkok, Thailand; 3 National Nanotechnology Center (NANOTEC), National Science and Technology Development Agency (NSTDA), Pathum Thani, Thailand; 4 Learning Institute, King Mongkut’s University of Technology Thonburi, Bangkok, Thailand; 5 Department of Mathematics, Faculty of Science, King Mongkut’s University of Technology Thonburi, Bangkok, Thailand; 6 King Mongkut’s University of Technology Thonburi, Ratchaburi Campus, Ratchaburi, Thailand; University of Illinois at Urbana-Champaign, UNITED STATES

## Abstract

Honeybees (*Apis mellifera*) play a significant role in the pollination of various food crops and plants. In the past decades, honeybee management has been challenged with increased pathogen and environmental pressure associating with increased beekeeping costs, having a marked economic impact on the beekeeping industry. Pathogens have been identified as a contributing cause of colony losses. Evidence suggested a possible route of pathogen transmission among bees via oral-oral contacts through trophallaxis. Here we propose a model that describes the transmission of an infection within a colony when bee members engage in the trophallactic activity to distribute nectar. In addition, we examine two important features of social immunity, defined as collective disease defenses organized by honeybee society. First, our model considers the social segregation of worker bees. The segregation limits foragers, which are highly exposed to pathogens during foraging outside the nest, from interacting with bees residing in the inner parts of the nest. Second, our model includes a hygienic response, by which healthy nurse bees exterminate infected bees to mitigate horizontal transmission of the infection to other bee members. We propose that the social segregation forms the first line of defense in reducing the uptake of pathogens into the colony. If the first line of defense fails, the hygienic behavior provides a second mechanism in preventing disease spread. Our study identifies the rate of egg-laying as a critical factor in maintaining the colony’s health against an infection. We propose that winter conditions which cease or reduce the egg-laying activity combined with an infection in early spring can compromise the social immunity defenses and potentially cause colony losses.

## Introduction

Agricultural productivity depends greatly on both wild and managed pollinators [1]. It was estimated that insect pollinators contributed to the economic value of crop production around 153 billion euros worldwide (2005 estimate) [2]. However, large-scale losses of managed honeybee colonies have been reported in some parts of North America and Europe in the past decades [3–7]. Additionally, in 2006, a special case of collapse in which adult worker bees rapidly disappear from colonies, leaving a large amount of brood to die, was widely observed in the US. This phenomenon is referred to as Colony Collapse Disorder (CCD), and its exact causes remain unclear [3, 8]. Although the global number of managed colonies has risen by about 30% since 2000 [9], honeybee management has been challenged with increased pathogen and environmental pressure associating with increased beekeeping costs.

The causes of colony losses are attributed to multiple possible factors among emerging pathogens and pests, reduced genetic diversity, the use of pesticides, shortage of high-quality food, and environmental changes [4, 6, 8, 10]. Important pathogens and pests known to contribute to colony losses include *Paenibacillus larvae* and *Melissococcus plutonius* (causative agents for American and European foulbrood, respectively), parasitic mite *Varroa destructor*, parasitic microsporidia *Nosema* species, and several honeybee viruses [10, 11].

Honeybees living in large colonies are prone to the rapid spreading of pathogens among individuals due to high population density and high contact rates. Trophallaxis (mouth-to-mouth food sharing) is considered a routine behavior that facilitates pathogen transmission [12, 13]. For example, trophallaxis may be the predominant mechanism of horizontal viral transmission. Nurse bees infected with viruses can transmit them to the larvae via trophallaxis since they rely heavily on nurses’ tending and feeding. The transmission route is supported by the detection of viruses, such as deformed wing virus (DWV) [14], sacbrood virus (SBV) [15], and Israeli acute paralysis virus (IAPV) [16], in larval food. In addition, the detection of viruses in the hypopharyngeal gland of infected worker bees was demonstrated for acute bee paralysis virus (ABPV) [17], SBV [18], and IAPV [16], implying a possible foodborne transmission route driven by trophallaxis.

Under pathogen pressure, honeybee colonies have developed several mechanisms to prevent disease transmission. For instance, structured interactions between honeybee members and their adaptive behaviors induced upon infection can reduce the impact of infectious diseases at the colony level. The mechanism is collectively known as ‘social immunity’ [19–23], including, for example, spatial separation of high-risk bees from low-risk bees [24, 25], decrease in contacts with infected bees [26], cleaning the body surface of nest-mates to remove foreign material by allogrooming [27], self-removal of infected bees [28], removal of dead or infected brood [29–31], and removal of infected nest-mates [32, 33].

The population dynamics within a honeybee colony has been studied extensively using mathematical models. They provide insightful understandings of the potential mechanisms and various factors influencing colony growth and death. Martin [34] developed a model that considered the demographic structure of the honeybee colony by dividing honeybee members into compartments of different ages. The model then incorporated the effects of DWV and ABPV along with their vector mites *V. destructor*. Together with another subsequent model [35], the authors identified a threshold number of mites carrying DMV or ABPV that could potentially kill the colony. Eberl et al. [36] studied a model of interactions between honeybees, *V. destructor*, and ABPV, and identified a threshold number of worker bees required to maintain the brood-rearing activity. The number of worker bees below the threshold put the colony at risk of collapse.

Khoury et al. [37] developed a model to investigate the forager death rate as a key factor driving the colony to failure. They proposed that as stressors elevate the forager death rate, worker bees at younger ages are recruited to forage. These young foragers have a higher risk of death during foraging than mature foragers, which accelerates the recruitment of even younger worker bees to forage, and depletes the worker population to the point where brood rearing cannot be maintained. A subsequent model by the same group [38] incorporated food availability as another key factor in the brood-rearing activity, and the prediction of the models was experimentally demonstrated in a following work [39]. Booton et al. [40] extended Khoury’s model frameworks to include density-dependent mortality of worker bees. The model predicted that a small change in the rate of regulatory processes such as forager recruitment, social inhibition, and egg-laying could cause sudden colony losses due to a critical transition via a saddle-node bifurcation.

Betti et al. [41] constructed a model to predict how the onset of an infection in relation to the onset of winter can determine the loss of the colony. The model was later expanded to incorporate an age structure of the worker bees [42]. These models link the dynamics of pathogenic infections inside the colony with the dynamics of the honeybee population to explain colony losses. Petric et al. [43] combined the effects of *N. ceranae* infection and elevated forager losses due to external stressors into a model and concluded that the combined effect might lead to colony death. Modeling inter-colony pathogen transmission has also been presented. Bartlett et al. [44] examined how management practices (e.g., colony numbers and colony arrangement configurations) at the apiary scale could impact pathogen prevalence.

In the present work, we develop a model focusing on disease transmission among bee members primarily via trophallactic behaviors. An infection is introduced into the colony when a foraging bee becomes infectious outside the nest and transmits it while unloading nectar via trophallaxis to a nectar-receiver. Nectar-receivers then spread the infection to bees of other classes, including nurses and, subsequently, larvae. Our model is generally applicable to pathogens transmitted via a foodborne transmission pathway (i.e., presumably driven by trophallaxis).

The present model has several key features that are different from other previous models. In previous models, the role of social immunity, particularly the social segregation between low-risk and high-risk bees and the hygienic behavior toward sick bees, has not been the main focus before (except a couple of models, see [24, 45]). We incorporate the two features into our model. First, our model implements social segregation. High-risk individuals such as foragers are limited to contact only nectar-receivers, but not other vulnerable individuals (nurses and brood) inside the nest. Second, our model includes the hygienic behavior, by which healthy nurses actively remove infected workers and brood from the colony. The two features constitute the social immunity in our model and play a crucial role in preventing horizontal transmission of pathogens from the infected ones to other nest-mates.

Additionally, previous models were deterministic-based (except [24] and [44]), neglecting the stochastic nature of infection transmission. Our study emphasizes the role of stochasticity that contributes to colony status. The conditions that do not cause colony death in a deterministic setting can cause colony mortality in our stochastic simulations.

We identify the rate of egg-laying as a critical factor enabling effective hygienic behavior of the colony and determining the survival of the colonies in the face of a pathogen. We demonstrate conditions in which colonies prevent the disease from transmission within the nest and in which colonies experience losses. Finally, our model also provides a possible explanation of massive and widespread colony losses in early spring, a phenomenon known as “spring dwindling.”

## Results and discussion

Our model is based on two important aspects of social immunity in honeybee colonies. First, the model considers the social segregation of worker bees based on their tasks. Thereby, we separate worker bees into three classes, namely, nurses, nectar-receivers, and foragers. Nurse bees are mainly responsible for brood rearing, including inspecting brood cells and feeding larvae [46, 47]. Foragers, which are highly exposed to pathogens during foraging outside the nest, only interact with nectar-receivers but do not directly interact with nurse bees and brood [48]. These social and spatial segregations between individuals with a high risk of infection and vulnerable individuals inside the hive are believed to limit disease spread at the colony level [20].

Another important aspect of our model is to implement a hygienic social response of healthy workers toward infected bees. The removal of infected brood from their cells has been demonstrated for many brood pathogens, including *P. larvae* [30], *Ascosphaera apis* (pathogen for chalkbrood disease) [49], SBV [50], and *V. destructor* [51]. In addition, worker bees have been shown to remove worker nest-mates infected with DWV [32] and *N. ceranae* [33]. The recognition of infected individuals by bees performing hygienic behavior is triggered by olfactory cues, e.g., by detecting cuticular hydrocarbon changes in sick bees [32, 52]. Here, we include in our model the hygienic behavior by which healthy nurse bees 1) remove infected hive-mates, and 2) remove infected brood from the population.

### Core model

**Fig 1:**
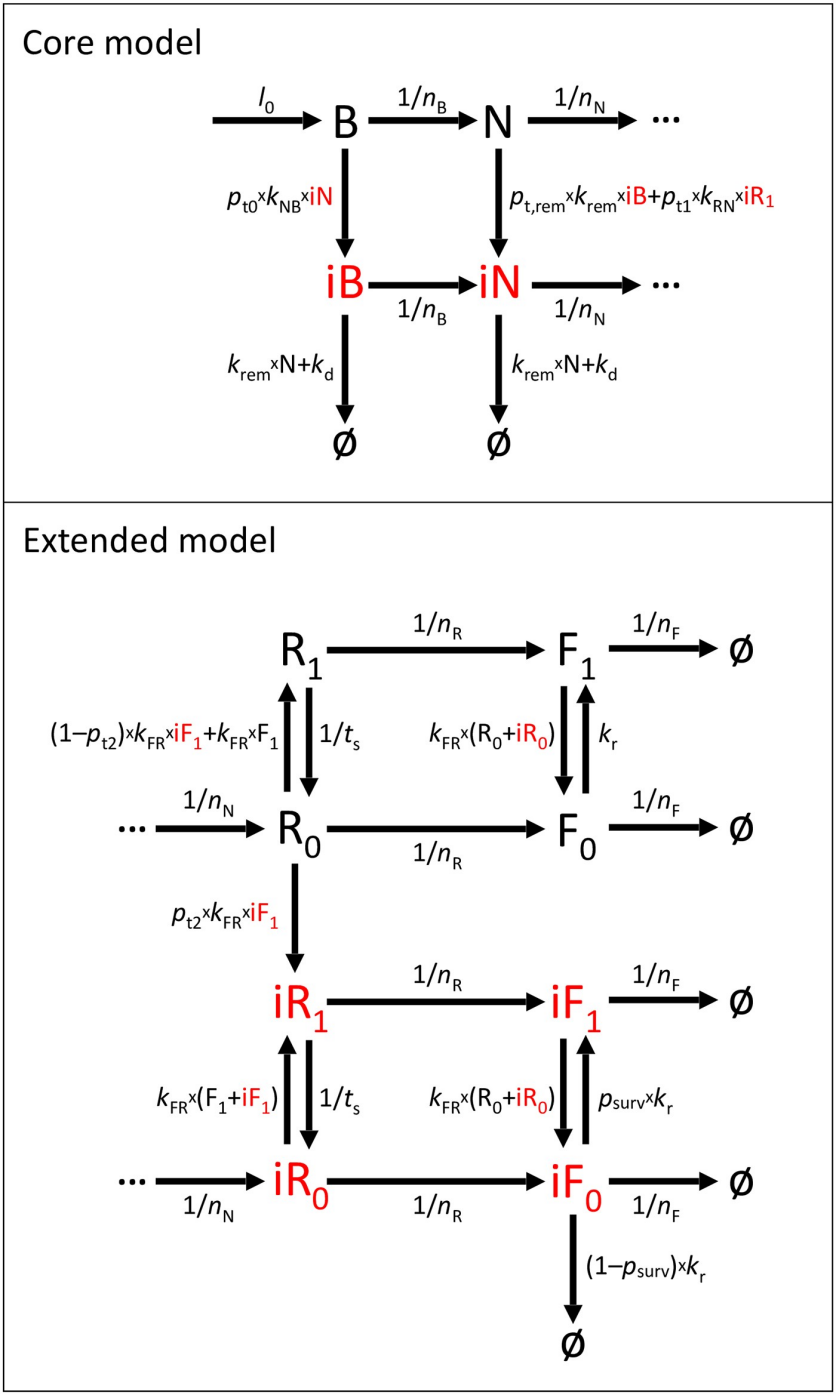
Model diagram. Upper: Core model. Lower: Extended model. The diagram illustrates class (horizontal arrows) and state (vertical arrows) transitions of honeybees. The queen produces new brood (B). As bees are older, they change their classes from brood (B), nurses (N), nectar-receivers (R_0_ and R_1_), to foragers (F_0_ and F_1_). Bees can change into an infection state (iB, iN, iR_0_, iR_1_) with a certain probability if they receive nectar from infected ones. Nectar-receivers and foragers can also change their nectar-loaded state between unloaded (subscript 0) and loaded (subscript 1). *φ* represents ‘death’. See the main text for a more detailed description of each transition. The full model is composed of both core and extended parts.

We first construct a core model consisting of only brood and nurses (Fig 1, upper panel and ordinary differential equations in Eqs 1–4), where B, iB, N and iN represent the number of healthy brood, infected brood, healthy nurses, and infected nurses, respectively.

$$\begin{array}{c} {\text{B}}^{\prime }=l_{0}-\frac{\text{B}}{n_{\text{B}}}-p_{\text{t0}}\cdot k_{\text{NB}}\cdot \text{iN}\cdot \text{B} \end{array}$$ (1)

$$\begin{array}{c} {\text{iB}}^{\prime }=-\frac{\text{iB}}{n_{\text{B}}}+p_{\text{t0}}\cdot k_{\text{NB}}\cdot \text{iN}\cdot \text{B}-k_{\text{rem}}\cdot \text{iB}\cdot \text{N}-k_{\text{d}}\cdot \text{iB} \end{array}$$ (2)

$$\begin{array}{c} {\text{N}}^{\prime }=\frac{\text{B}}{n_{\text{B}}}-\frac{\text{N}}{n_{\text{N}}}-p_{\text{t1}}\cdot k_{\text{RN}}\cdot {\text{iR}}_{1}\cdot \text{N}-p_{\text{t,rem}}\cdot k_{\text{rem}}\cdot \text{iB}\cdot \text{N} \end{array}$$ (3)

$$\begin{array}{c} {\text{iN}}^{\prime }=\frac{{\text{B}}_{\text{i}}}{n_{\text{B}}}-\frac{\text{iN}}{n_{\text{N}}}+p_{\text{t1}}\cdot k_{\text{RN}}\cdot {\text{iR}}_{1}\cdot \text{N}-k_{\text{rem}}\cdot \text{iN}\cdot \text{N}+p_{\text{t,rem}}\cdot k_{\text{rem}}\cdot \text{iB}\cdot \text{N}-k_{\text{d}}\cdot \text{iN} \end{array}$$ (4)

The above equations are based on the following assumptions:

1. Brood is produced with an egg-laying rate constant *l*_0_. Healthy and infected brood develop into healthy and infected nurses, respectively, with a rate equal to 1/*n*_B_, where *n*_B_ = 20 days [53]. Healthy and infected nurses develop into healthy and infected nectar-receivers, respectively, with a rate equal to 1/*n*_N_, where *n*_N_ = 10 days [53]. Nectar-receivers and foragers are not considered in the core model but will be included in an extended model described below.
2. Upon feeding, infected nurses transmit the infection to brood. We assume brood gets infected with a rate *p*_t0_ ⋅ *k*_NB_ ⋅ iN, where *p*_t0_ is a probability of infection transmission per contact between an infected nurse and a brood, *k*_NB_ is a rate of contact between nurses and brood, and iN is the number of infected nurses (Eqs 1 and 2). Here, we assume brood representing eggs, larvae, and pupae, all together for simplicity.
3. Healthy nurses remove infected hive-mates from the colony, which is carried out as healthy nurses either killing or chasing away infected ones. We assume in our model that healthy nurses (N) actively remove infected nurses (iN) from the population with a constant rate *k*_rem_ (Eq 4).
4. Healthy nurses (N) actively remove infected brood (iB) from their cells, also with a constant rate *k*_rem_ (Eq 2). Although it was reported that the brood removal behavior was performed by middle-aged bees (age 15–18 days) [29], there was also evidence that nurses bee participate in the activity as well [54]. Here we assume that nurse bees can remove both infected brood and workers.
5. Nurses become infected upon contacting infected nectar-receivers, as Gruter and Farina [55] observed that nectar-receivers offer nectar-transferring contacts to nurse bees during the trip to store nectar inside the hive. We assume the infection occurs with a rate *p*_t1_ ⋅ *k*_RN_ ⋅ iR_1_, where *p*_t1_ is a probability of infection transmission per contact between an infected nectar-receiver and a nurse, *k*_RN_ is a rate of contact between nectar-receivers and nurses, and iR_1_ is the number of infected nectar-receivers who carry nectar, which will be described further in an extended model. In the core model, we treat *p*_t1_ ⋅ *k*_RN_ ⋅ iR_1_ in Eqs 3 and 4 as a constant, which is equal to 5 × 10^−4^ per day.
6. Nurses do not get infected from other infected nurses. As we assume nectar distribution as a major route of infection transmission, we ignore infection transmission between nurse bees.
7. An infection is assumed to spread in only one direction from nectar-donors to nectar-receivers.
8. During infected brood removal by hygienic bees, workers performing the removal may become infected by handling contaminated tissues with a probability *p*_t,rem_ (Eqs 3 and 4). The default value of *p*_t,rem_ is 0.0 and we will explore this parameter later.
9. Infection of individual honeybees results in variable outcomes depending on the infection loads and other stress factors. For example, most viruses infect honeybees without clinical symptoms [56], but the infection can become acute following other stresses, leading to honeybee mortality. We set the default value of the disease-related death rate *k*_d_ = 0.0 per day to represent subclinical infection. Later, we will vary the parameter to explore for lethal infection effects on the colony status. (We assume both infected brood and workers have the same death rate, *k*_d_, for simplicity (Eqs 2 and 4)).

**Fig 2:**
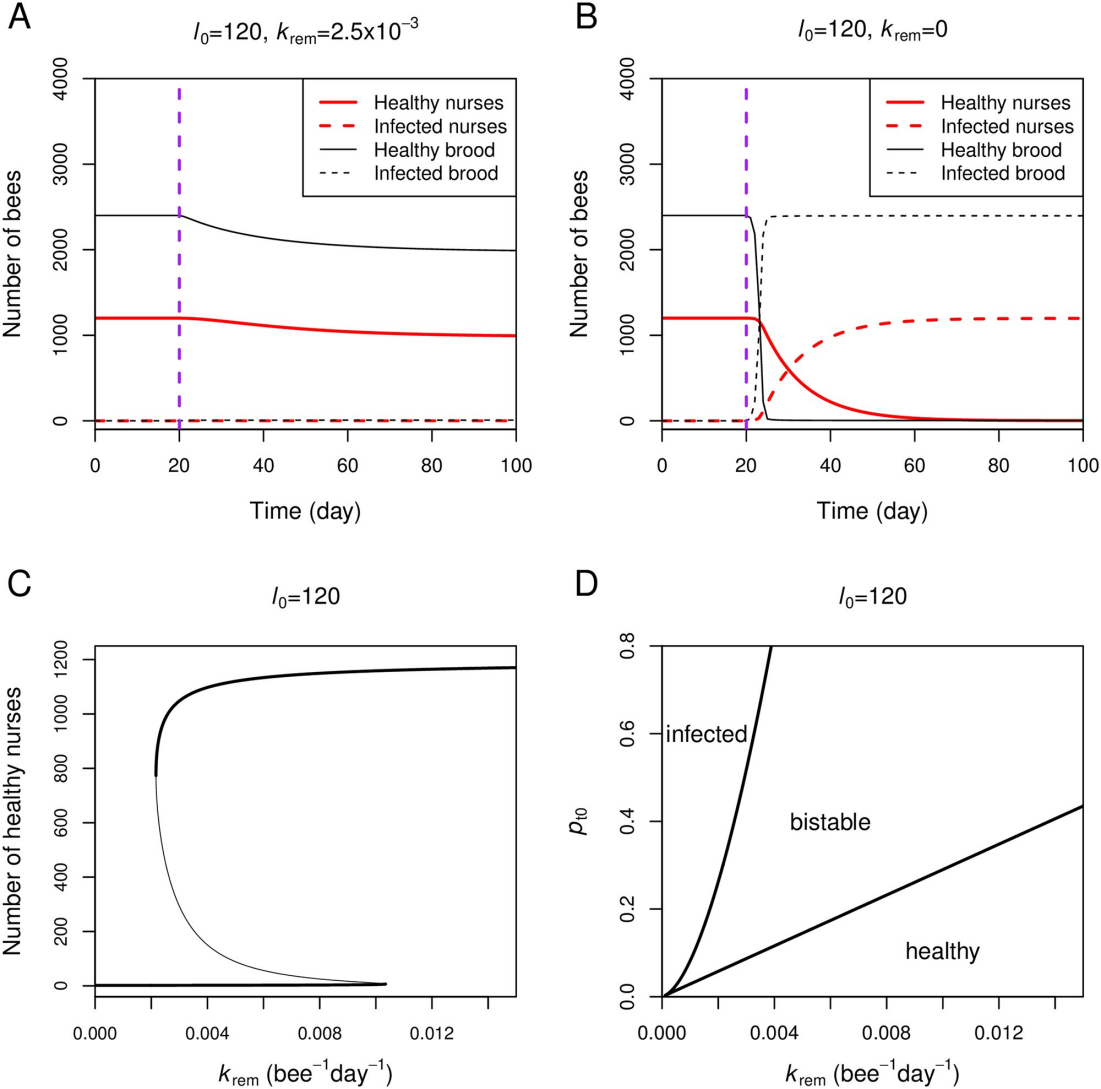
Role of the hygienic behavior (*k*_rem_). A: Healthy brood and nurses in a colony with the hygienic behavior are maintained against an infection. The simulation is based on Eqs 1–4 with model parameters listed in Table 1 with *l*_0_ = 120 and *k*_rem_ = 2.5 × 10^−3^. We treat *p*_t1_ ⋅ *k*_RN_ ⋅ iR_1_ as a parameter, which is equal to 5 × 10^−4^. The infection is introduced (*p*_t1_ ⋅ *k*_RN_ ⋅ iR_1_ = 5 × 10^−4^) at day 20 (vertical purple line). B: All brood and nurses in a colony without the hygienic behavior become infected. The simulation setting is the same as in panel A, except that *k*_rem_ = 0. C: The steady-state number of healthy nurses is plotted as *k*_rem_ is varied while other parameters are fixed. Bold lines represent a stable steady-state and a thin line represents an unstable steady-state. D: The 2-parameter bifurcation diagram is plotted as both *k*_rem_ and *p*_t0_ are varied. The diagram is divided into three regions depending on the state of the colony (healthy, bistable, and infected).

With model parameters listed in Table 1, the equations are solved numerically with XPPAUT (http://www.math.pitt.edu/~bard/xpp/xpp.html). Fig 2A and 2B compare dynamics of the colony exhibiting and not exhibiting the hygienic behavior (*k*_rem_ = 0.0025 and 0, respectively). In Fig 2A, a colony with the hygienic behavior is shown to prevent disease transmission. In contrast, the entire bee population in a colony without the hygienic behavior (Fig 2B) quickly becomes infected after an infection is introduced.

**Table 1:** Model parameters.

Parameter	Default value	Description	Reference
*l*_0_	50–2000 eggs/day	Egg-laying rate	[37] and variable
*n*_B_	20 days	Days in brood class	[53]
*n*_N_	10 days	Days in nurse class	[53]
*n*_R_	11 days	Days in nectar-receiver class	[53]
*n*_F_	14 days	Days in forager class	[53]
*k*_NB_	0.1 (bee ⋅ day)^–1^	Contact rate between nurse bees and brood	calculated from [47] (S1 Text)
*k*_RN_	0.5 (bee ⋅ day)^–1^	Contact rate between nectar-receiver and nurse bees	calculated from [55] & [57] (S1 Text)
*k*_FR_	1.44 (bee ⋅ day)^–1^	Contact rate between foragers and nectar-receivers	calculated from [58] (S1 Text)
*t*_s_	0.01 day	Duration of a storage cycle	[57]
*k*_r_	0.5 (day)^–1^	Rate of nectar-collecting	variable
*p*_t0_, *p*_t1_, *p*_t2_	0.3	Probability of disease transmission per contact	variable
*p*_surv_	0.0	Probability of infected foragers to return home	variable
*k*_rem_	2.5 × 10^−3^ (bee ⋅ day)^–1^	Rate of infected bee removal by healthy nurses	variable
*p*_t,rem_	0.0	Probability of healthy nurses being infected from infected brood during performing hygienic removal	variable
*k*_d_	0.0 (day)^–1^	Disease-related death rate of infected bees	variable

Bigio [31] observed that the levels of hygienic brood removal behavior are considerably varied from one colony to another. Therefore, in Fig 2C, we vary the value of *k*_rem_ while fixing other model parameters and plot the steady-state number of healthy nurse bees. With the parameter set listed in Table 1, the diagram predicts that different levels of the hygienic behavior lead to different colony fates. With small values of *k*_rem_ below a threshold at the left bifurcation point (*k*_rem_ = 0.0021), the infection can spread very quickly owing to a positive feedback loop between infected nurses and infected brood. Since eclosed nurses who are infected as brood participate in rearing the next cohort of nurses, infection from infected nurses to brood results in the positive feedback loop. At values of *k*_rem_ larger than the threshold, a bistable switch between healthy and infected nurses emerges from a double-negative feedback loop between the two nurse states. Healthy nurses keep inspecting infected ones and removing them, pushing the dynamics toward a healthy colony state. Infected nurses transmit the infection to brood, turning them into infected nurses and pushing the dynamics toward a colony’s health-compromised state. *k*_rem_ values above the second threshold at the right bifurcation point always maintain the healthy colony state against the infection. The two-parameter bifurcation diagram in Fig 2D reveals a relationship between the value of *k*_rem_ and *p*_t0_. As the probability of brood infection (*p*_t0_) increases (e.g., disease is more transmissible), a larger value of *k*_rem_ is required to keep the colony in a healthy state.

**Fig 3:**
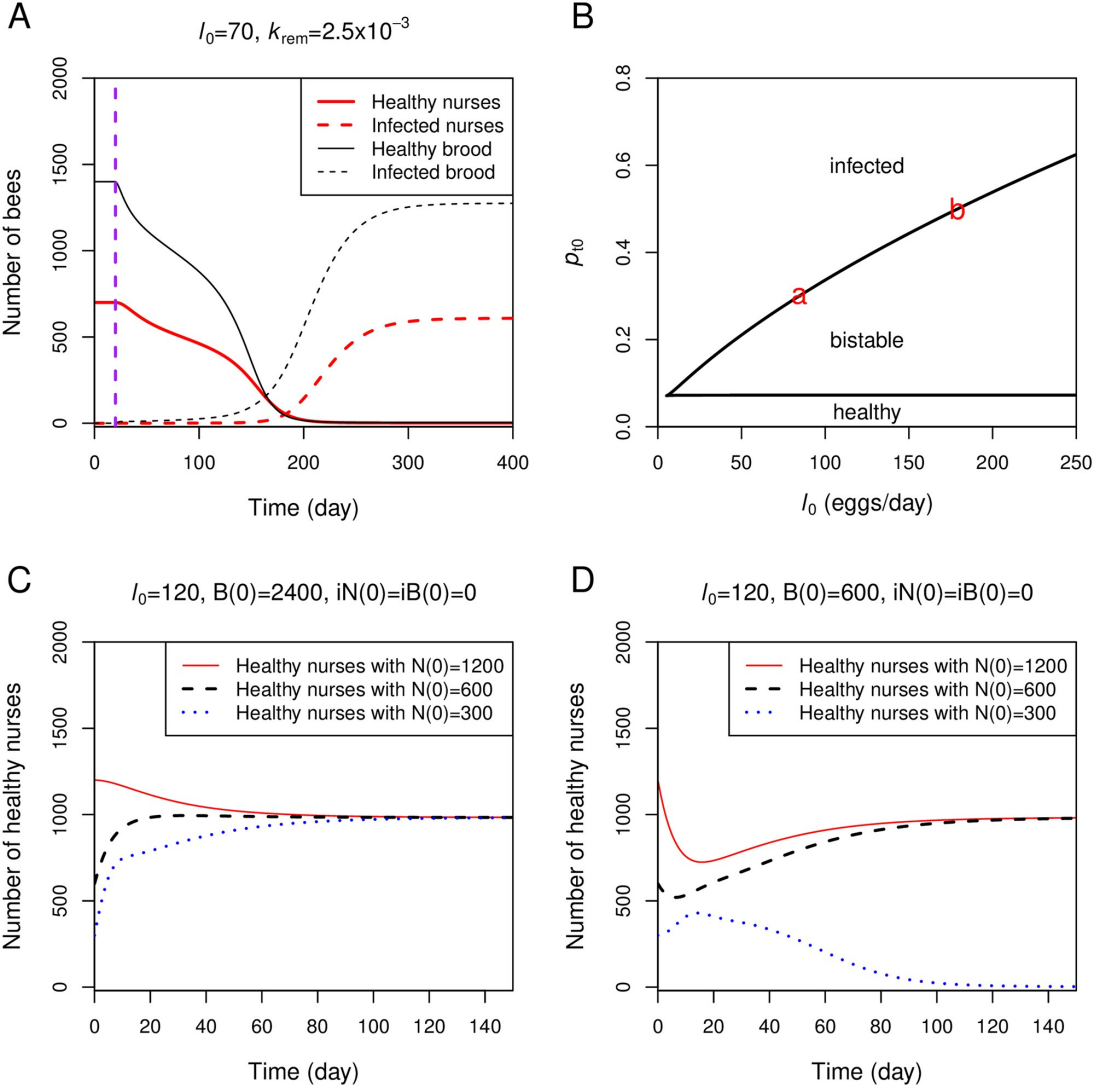
Role of the egg-laying rate (*l*_0_). A: All brood and nurses in a small-size colony (e.g., colony with a low egg-laying rate) become infected even with the hygienic behavior response. The simulation setting is the same as in Fig 2A, except that *l*_0_ = 70. The infection is introduced (*p*_t1_ ⋅ *k*_RN_ ⋅ iR_1_ = 5 × 10^−4^) at day 20 (vertical purple line). B: The two-parameter bifurcation diagram is plotted between *l*_0_ and *p*_t0_ while other parameters are fixed. The red letters a and b mark the critical values of *l*_0_, below which the colonies are always infected when *p*_t0_ is 0.3 and 0.5, respectively. C: Simulation of three colonies with the same parameter set from Fig 2A, but the simulation begins with 2400 initial healthy brood and different initial numbers of healthy nurses. The infection is introduced (*p*_t1_ ⋅ *k*_RN_ ⋅ iR_1_ = 5 × 10^−4^) at day 0. D: Simulation setting is the same as that of panel C, except that the initial number of healthy brood is 600.

Fig 3 shows that the egg-laying rate (*l*_0_) is another important factor determining the infection state of the colony. When *l*_0_ is reduced from 120 to 70, the entire bee population becomes infected even when the hygienic behavior is in place (Fig 3A). To explore how the parameter *l*_0_ affects the infection state of the colony, Fig 3B plots a two-parameter bifurcation diagram between *l*_0_ and *p*_t0_. At the transmission probability (*p*_t0_) equal to 0.3, there is a critical value of *l*_0_ around 84, below which all bees become infected (red letter a in Fig 3B). Above the critical value of *l*_0_, the system exhibits bistability, where the colony state depends on an initial condition of the colony, for example, healthy colonies remain healthy under the face of an infection. The figure also reveals that larger colonies (e.g., colonies with high laying rates) can cope with more transmissible infection. For example, a colony with *l*_0_ around 180 can prevent the spread of infection with *p*_t0_ up to 0.5 (red letter b in Fig 3B). Our results demonstrate that the hygienic response is enhanced in large-size colonies.

The presence of the bistable region in Fig 3B also implies that the colony state under an infection is subject to the current numbers of brood and nurses. If a colony begins with a sufficient amount of either healthy nurses or brood, it can maintain its healthy state. In Fig 3C, a sufficient number of healthy brood (B(0) = 2400), which subsequently converts into healthy nurses, maintains the colony in the uninfected state even when the number of healthy nurses is initially small. In Fig 3D, a sufficient number of healthy nurses can maintain the colony in a healthy state even when the number of healthy brood is initially small (B(0) = 600). However, when the numbers of both healthy brood and nurses are initially small, the whole colony becomes infected. Our results imply that any stressors that perturb the honeybee population in such a way that reduces the numbers of brood and nurses may cause the whole colony to become infected under an infection.

**Fig 4:**
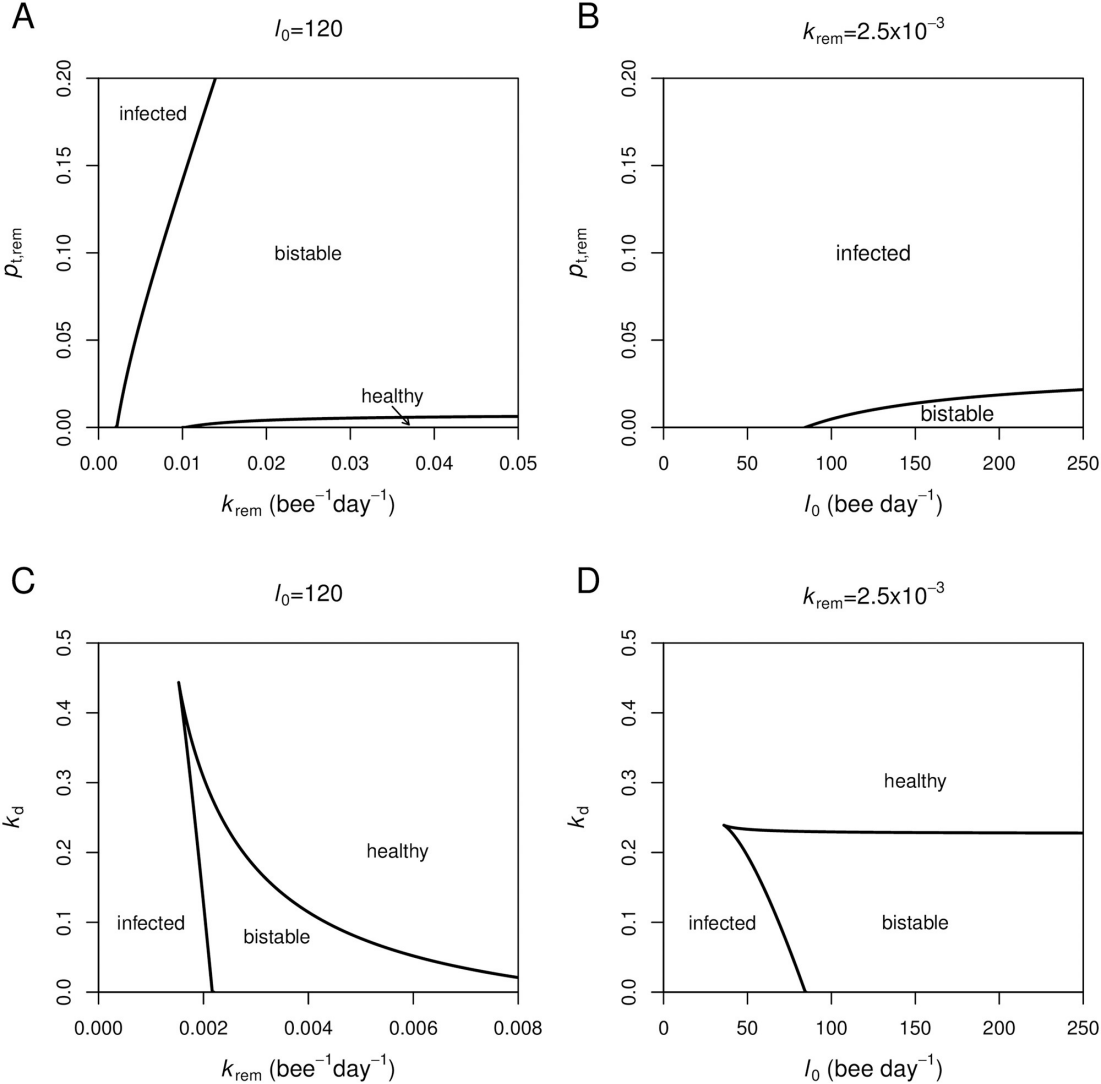
Role of the probability of nurses being infected from infected brood (*p*_t,rem_) and the death rate of infected bees (*k*_d_). The two-parameter bifurcation diagrams are plotted between A: *k*_rem_ and *p*_t,rem_, B: *l*_0_ and *p*_t,rem_, C: *k*_rem_ and *k*_d_, and D: *l*_0_ and *k*_d_. The diagrams are divided into regions corresponding to the state of the colony (healthy, bistable, and infected).

In Fig 4, we explore two more parameters in our model, namely the probability of nurses being infected from infected brood during performing hygienic removal (*p*_t,rem_) and the death rate of infected bees (*k*_d_). The two-parameter bifurcation diagrams in Fig 4A and 4B reveal that as *p*_t,rem_ increases, larger values of *k*_rem_ and *l*_0_ are needed in maintaining the colony’s health against an infection. In Fig 4C and 4D, *k*_d_ shows an opposite effect. As the death rate of infected bees increases, smaller values of *k*_rem_ and *l*_0_ are required to maintain the colony in a healthy state. When the death rate becomes very high, the infection rapidly kills the infected bees, preventing further transmission to other nest-mates, and the colony is always in a healthy state.

### Extended model

$$\begin{array}{c} {\text{R}}_{0}^{\prime }=\frac{\text{N}}{n_{\text{N}}}-\frac{{\text{R}}_{0}}{n_{\text{R}}}-k_{\text{FR}}\cdot \left({\text{F}}_{\text{1}}+{\text{iF}}_{\text{1}}\right)\cdot {\text{R}}_{0}+\frac{{\text{R}}_{1}}{t_{\text{s}}} \end{array}$$ (5)

$$\begin{array}{c} {\text{R}}_{1}^{\prime }=-\frac{{\text{R}}_{1}}{n_{\text{R}}}+k_{\text{FR}}\cdot \left({\text{F}}_{\text{1}}+\left(1-p_{\text{t2}}\right)\cdot {\text{iF}}_{\text{1}}\right)\cdot {\text{R}}_{0}-\frac{{\text{R}}_{1}}{t_{\text{s}}} \end{array}$$ (6)

$$\begin{array}{c} {\text{iR}}_{0}^{\prime }=\frac{\text{iN}}{n_{\text{N}}}-\frac{{\text{iR}}_{0}}{n_{\text{R}}}-k_{\text{FR}}\cdot \left({\text{F}}_{\text{1}}+{\text{iF}}_{\text{1}}\right)\cdot {\text{iR}}_{0}+\frac{{\text{iR}}_{1}}{t_{\text{s}}} \end{array}$$ (7)

$$\begin{array}{c} {\text{iR}}_{1}^{\prime }=-\frac{{\text{iR}}_{1}}{n_{\text{R}}}+k_{\text{FR}}\cdot \left({\text{F}}_{\text{1}}+{\text{iF}}_{\text{1}}\right)\cdot {\text{iR}}_{0}+p_{\text{t2}}\cdot k_{\text{FR}}\cdot {\text{iF}}_{\text{1}}\cdot {\text{R}}_{0}-\frac{{\text{iR}}_{1}}{t_{\text{s}}} \end{array}$$ (8)

$$\begin{array}{c} {\text{F}}_{0}^{\prime }=\frac{{\text{R}}_{0}}{n_{\text{R}}}-\frac{{\text{F}}_{0}}{n_{\text{F}}}+k_{\text{FR}}\cdot \left({\text{R}}_{\text{0}}+{\text{iR}}_{\text{0}}\right)\cdot {\text{F}}_{1}-k_{\text{r}}\cdot {\text{F}}_{0} \end{array}$$ (9)

$$\begin{array}{c} {\text{F}}_{1}^{\prime }=\frac{{\text{R}}_{1}}{n_{\text{R}}}-\frac{{\text{F}}_{1}}{n_{\text{F}}}-k_{\text{FR}}\cdot \left({\text{R}}_{\text{0}}+{\text{iR}}_{\text{0}}\right)\cdot {\text{F}}_{1}+k_{\text{r}}\cdot {\text{F}}_{0} \end{array}$$ (10)

$$\begin{array}{c} {\text{iF}}_{0}^{\prime }=\frac{{\text{iR}}_{0}}{n_{\text{R}}}-\frac{{\text{iF}}_{0}}{n_{\text{F}}}+k_{\text{FR}}\cdot \left({\text{R}}_{\text{0}}+{\text{iR}}_{\text{0}}\right)\cdot {\text{iF}}_{1}-k_{\text{r}}\cdot {\text{iF}}_{0} \end{array}$$ (11)

$$\begin{array}{c} {\text{iF}}_{1}^{\prime }=\frac{{\text{iR}}_{1}}{n_{\text{R}}}-\frac{{\text{iF}}_{1}}{n_{\text{F}}}-k_{\text{FR}}\cdot \left({\text{R}}_{\text{0}}+{\text{iR}}_{\text{0}}\right)\cdot {\text{iF}}_{1}+p_{\text{surv}}\cdot k_{\text{r}}\cdot {\text{iF}}_{0} \end{array}$$ (12)

When foragers return from nectar-collecting, they transfer the gathered nectar by trophallaxis to worker bees responsible for food processing (nectar-receivers) [59]. Most nectar-receivers store nectar immediately in honey cells, but some nectar-receivers distribute it to second-order receivers, who are mainly nurse bees [55]. In an extended model (Fig 1, lower panel, and Eqs 5–12), we include nectar-receivers and foragers as two more classes of bees. Receivers are assumed to be either unloaded (R_0_ and iR_0_) or loaded (R_1_ and iR_1_) with nectar. Foragers are assumed similarly (F_0_ and iF_0_ for unloaded, and F_1_ and iF_1_ for loaded). Healthy and infected nurses in the core model become healthy and infected unloaded nectar-receivers in the extended model, respectively. Nectar-receivers then become foragers with a rate equal to 1/*n*_R_, where *n*_R_ = 11 days [53]. Foragers stay in their class until the end of their lifespan (with a rate transition out of the foraging class equal to 1/*n*_F_, where *n*_F_ = 14 days [53]).

Unloaded foragers collect nectar and become loaded with a rate constant *k*_r_, which describes several processes together including recruitment, foraging, and returning to the nest. Foragers are assumed to get an infection outside the nest. To simulate the onset of an infection into the colony, we introduce one infected forager returning from nectar-collecting at time *t*_0_ (i.e., iF_1_(*t*_0_) = 1). At the delivery area, loaded foragers (F_1_ or iF_1_) unload nectar to unloaded receivers (R_0_ or iR_0_), with a constant rate *k*_FR_, which is a contact rate between foragers and nectar-receivers (Eqs 9–12). The foragers then become unloaded and wait for next recruitment. Evidence showed that infection can greatly reduce homing ability of the infected foragers compared to healthy foragers [60–62]. Therefore, we assume infected foragers have a probability of *p*_surv_ to return home during the foraging trip (Eq 12).

Unloaded nectar-receivers that receive nectar from foragers become loaded, also with a constant rate *k*_FR_ (Eqs 5–8). If a healthy nectar-receiver unloads nectar from an infected forager, the former may become infected with a probability *p*_t2_ (Eq 8). Loaded nectar-receivers store nectar in honey cells inside the nest and return to the delivery area as an unloaded state with a constant rate 1/*t*_s_. During the storage trip, nectar-receivers offer feeding contacts to multiple nurse bees [55]. Such an interaction between an infected nectar-receiver and a healthy nurse results in a disease transmission to the latter with a probability *p*_t1_ (Eqs 3 and 4 in the core model).

As in the core model, infection is assumed to spread only from nectar-donors to nectar-receivers. The full model is composed of Eqs 1–12, where the infection rate of N (*p*_t1_ ⋅ *k*_RN_ ⋅ iR_1_ ⋅ N) in Eqs 3 and 4 is now dependent on the number of infected loaded nectar-receivers (iR_1_) in Eq 8. We do not consider the disease-related death of infected receivers and foragers as in the full model we will focus on subclinical infection of the colonies (i.e., *k*_d_ = 0). All model parameters are listed in Table 1.

**Fig 5:**
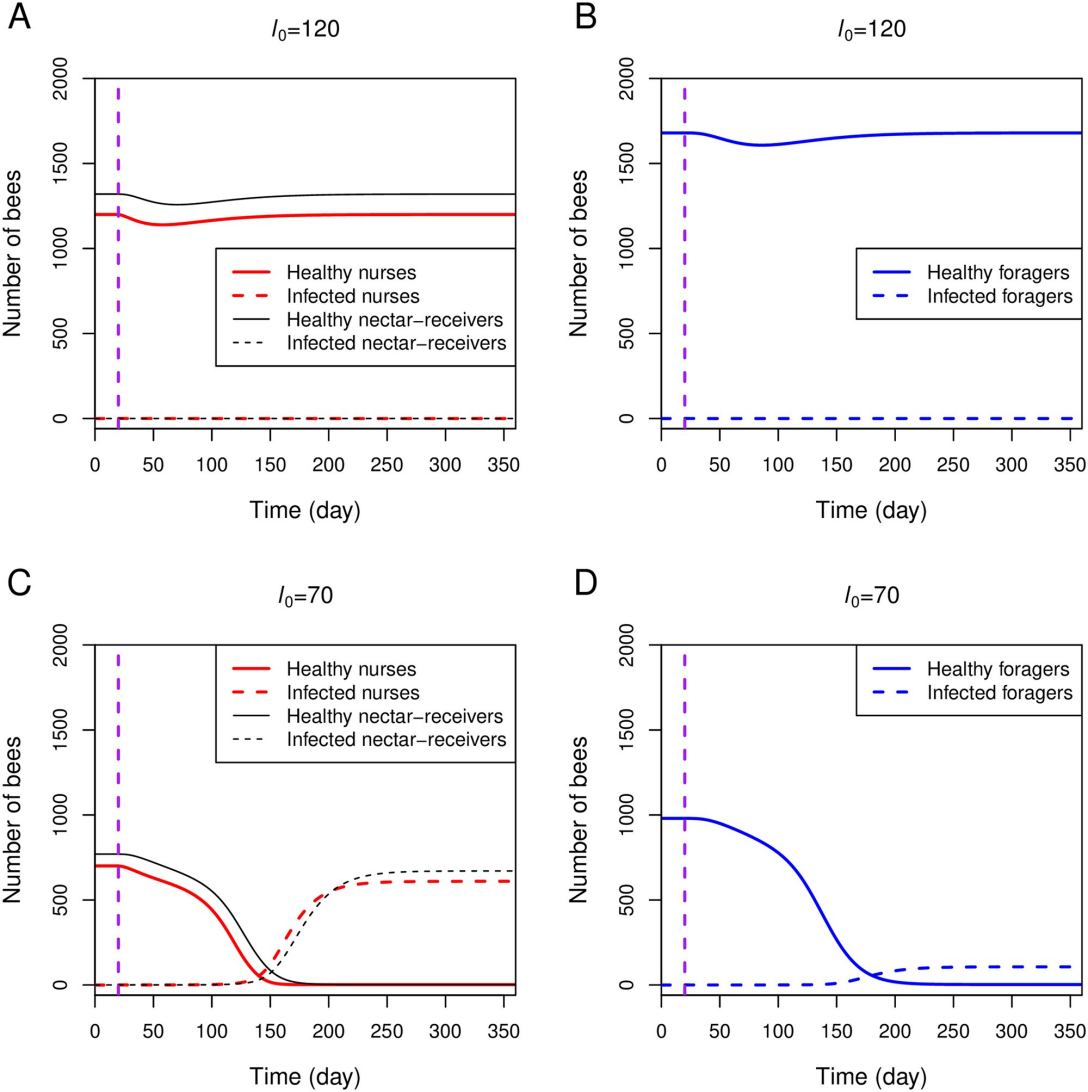
Simulations of the full model. The full model is simulated with parameters listed in Table 1 with *l*_0_ = 120 (panels A and B) and *l*_0_ = 70 (panels C and D). One infected forager returning from nectar-collecting (iF_1_) is introduced at day 20 (iF_1_(20) = 1) (vertical purple line).

Similar to the results shown in the core model, the laying rate influences the colony status in the full model. Colonies with large population sizes (e.g., colonies with high laying rates) maintain their healthy state (Fig 5A and 5B) while colonies with small population sizes become infected (Fig 5C and 5D) under an infection. It is evident from the simulation that once the colony makes a critical transition to the infected state, all hive bees become infected (Fig 5C). Therefore, all newly emerged foragers are also infected. If the homing ability of infected foragers is severely impaired (*p*_surv_ is small), our simulation in Fig 5C and 5D exhibits a condition where most of the foragers suddenly disappear from the hive, and the remaining hive bees are heavily infected.

Beekeepers often experience heavy colony losses over winter and early spring, a phenomenon known as “spring dwindling” [3]. When a honeybee colony enters the overwintering state, the queen halts producing eggs, and the number of bees declines toward the end of winter. We propose that this transiently small number of bees toward the end of winter, combined with the spread of disease inside the colony due to active nectar-distribution activity among honeybees in spring, may contribute to the colony losses. We simulate this situation by setting during winter months *l*_0_ = 0 (the queen ceases to produce eggs), *k*_r_ = 0 (all foragers stay at the hive), and *n*_N_, *n*_R_, and *n*_F_ are increased 4-folds (reflecting extended lifespan of winter bees [63]). Winter is assumed to last five months from day 210 to day 360 in the simulation.

**Fig 6:**
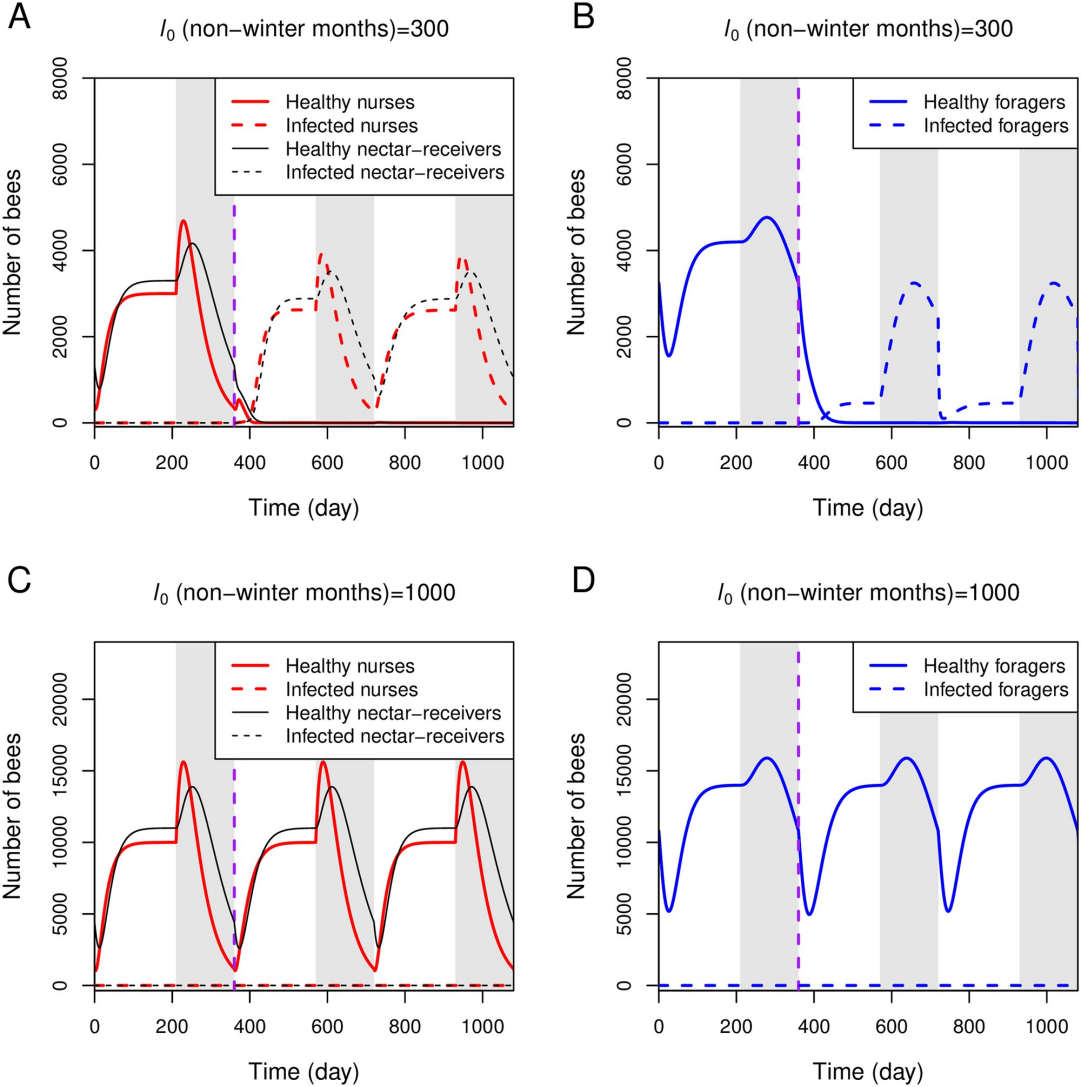
Simulations of the full model under seasonal effects. The full model is simulated with parameters listed in Table 1 under seasonal effects. During winter (grey stripes), which lasts five months a year, we set *l*_0_ = 0, *k*_r_ = 0, and *n*_N_, *n*_R_, and *n*_F_ are increased 4-folds. A and B: *l*_0_ during non-winter months = 300. C and D: *l*_0_ during non-winter months = 1000. One infected forager returning from nectar-collecting (iF_1_) is introduced at day 360 (vertical purple line).

Our simulation in Fig 6A and 6B demonstrates the dwindling of a small-size colony (*l*_0_ = 300 and 0 during non-winter and winter months, respectively). The egg-laying rate represents a colony of worker size around 10,500 bees and 6,000 brood in mid-summer (day 150) and 5,000 worker bees and broodless at the end of winter (day 360) under non-infection conditions. Under an infection introduced in early spring (day 360), when the colony population is small, the whole colony becomes infected and remains in the health-compromised state even when the number of honeybees rises later in late spring. As a result, all newly emerged foragers are also infected, and with impaired homing ability, they disappear while foraging. As we did not indicate precisely the point where the colony dies in our simulation, the simulation in Fig 6A and 6B continues until day 1080. However, we can reasonably assume that after all hive bees and foragers become infected, the colony mortality follows soon after as there are not enough foragers returning with food to maintain the colony’s growth and survival. As we set the death rate of infected bees (*k*_d_) equal to 0.0, our simulation demonstrates how subclinical infection leads to colony losses due to overwintering stresses.

Simulations with *l*_0_ = 1000 and 0 during non-winter and winter months, respectively, in Fig 6C and 6D, represent larger natural colonies. The colony has a worker size of around 35,000 bees and 20,000 brood in mid-summer (day 150) and 16,000 worker bees and ten brood at the end of winter (day 360) under non-infection conditions. Under the infection condition, our simulation shows that a honeybee colony of this size can prevent disease spread within the colony (Fig 6C and 6D), which again supports the impact of colony size (determined by the rate of egg-laying) on the social immunity response.

### Stochastic simulations

Deterministic simulations revealed colonies with large population sizes similar to what is observed in natural colonies could mitigate disease transmission risk within colonies. In this section, we translate our model into a stochastic counterpart to explore if the conditions that do not result in colony death in the deterministic simulation may cause mortality due to stochastic fluctuation in the number of bees. The stochastic model is based on the stochastic simulation algorithm (SSA) [64], and follows the method used in [65, 66]. Briefly, every term in the ODE-based model is considered as a stochastic event. The model then tracks the number of bees in each class and state as every event is simulated. More details of the model can be found in S2 Text.

**Fig 7:**
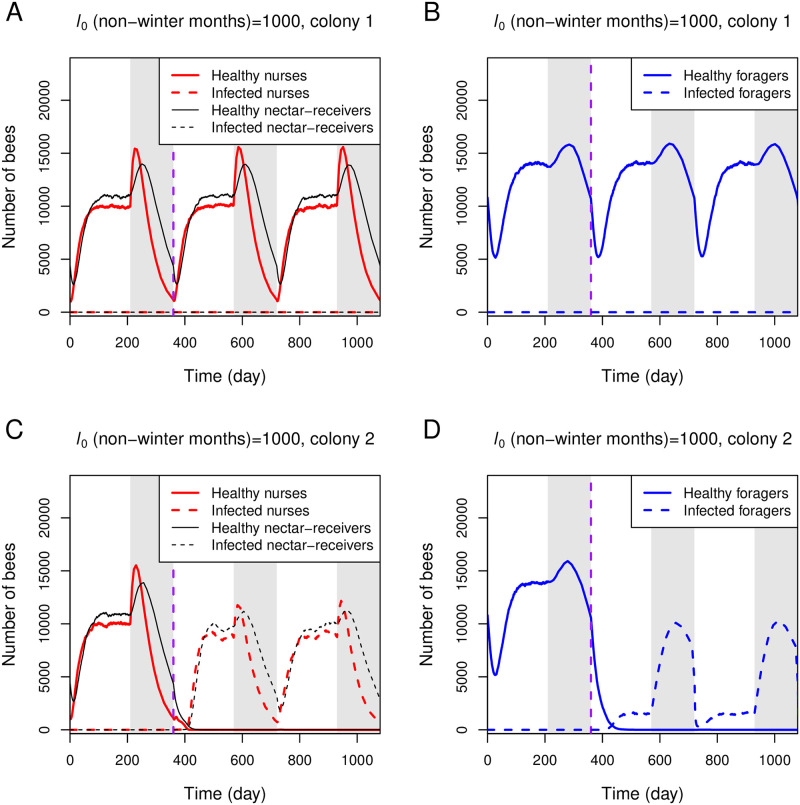
Stochastic simulations of the full model under seasonal effects. Stochastic simulations of two independent colonies under an identical condition are shown in upper and lower panels, respectively. Seasonal effects are implemented, as described in Fig 6 and the main text. Both colonies are simulated with the same parameter set (*l*_0_ during non-winter months = 1000 and other parameters from Table 1). One infected forager returning from nectar-collecting (iF_1_) is introduced at day 360 (vertical purple line).

Fig 7 illustrates two sample colonies under an identical condition (*l*_0_ during non-winter months = 1000 and other parameters in Table 1), in which one hive survives over two years after an infection (Fig 7A and 7B), while the other hive dies (Fig 7C and 7D). Our results suggest that two identical colonies exposed to an infection can have different fates due to inherent stochasticity within the colonies.

**Fig 8:**
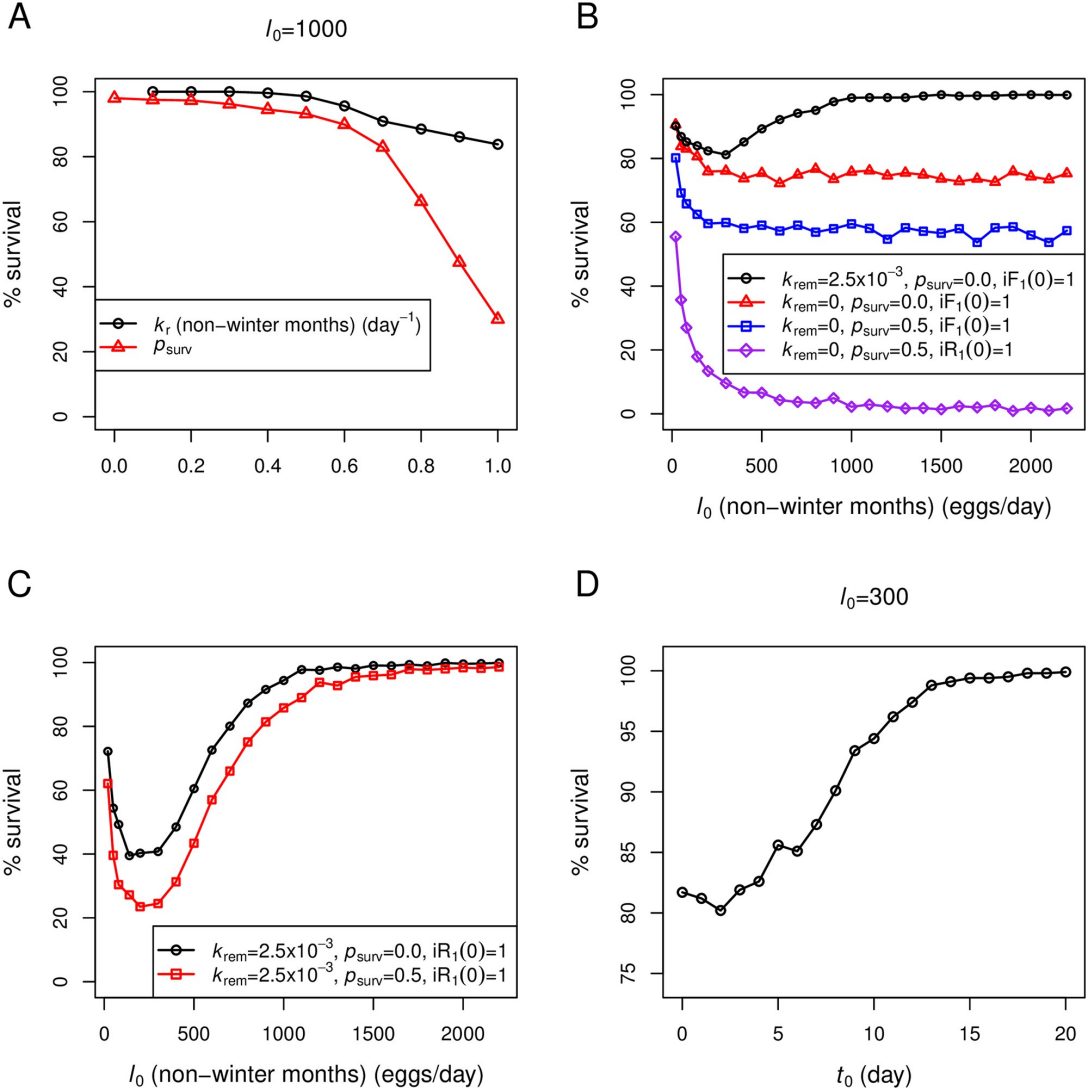
Percentages of survived colonies from stochastic simulations. Percentages of survived colonies are calculated from 1000 repeats of stochastic simulations. Model parameters are from Table 1, except those that are listed in each figure panel. Seasonal effects are implemented as described in the main text. A: *k*_r_ during non-winter months (black line) or *p*_surv_ (red line) is varied. B and C: *l*_0_ during non-winter months is varied. D: The timing of an infection (*t*_0_) is varied in relation to the onset of spring (i.e., iF_1_(*t*_0_) = 1).

In Fig 8, we vary parameters in our model and calculate the percentages of survived colonies from 1000 stochastic simulation repeats. Here, we identify a colony’s death when the proportion of infected hive bees (nurses and nectar-receivers) reaches 0.8 within one year after one infected forager returning from nectar-collecting (iF_1_) is introduced at the onset of spring. Fig 8A, black line, shows that the colony survival percentage decreases as the nectar-collecting rate (*k*_r_) increases. When more nectar is collected, higher trophallactic activity among honeybees within the colony leads to a higher probability of pathogen spread and colony losses. The percentage of colony survival also decreases as the homing ability of infected foragers (*p*_surv_) increases (Fig 8A, red line) as it allows infected foragers to make multiple rounds of nectar-unloading contacts, which increases the infection risk of nectar-receivers. Thus, the impaired homing ability of infected foragers may be considered an adaptive behavior of individual bees to limit the infection spread. However, impaired homing ability of infected foragers may also induce the premature transition of hive bees to foragers that accelerates the depletion of the population pool as proposed in [37–39]. Note that we did not implement the premature transition mechanism in the current version of our model as this behavior has already been a subject of investigation in many previous studies.

Fig 8B shows the percentages of survived colonies as *l*_0_ during non-winter months is varied from 50 to 2200 (*l*_0_ during winter months is 0). Simulations in the figure reveal patterns that cannot be predicted by the deterministic model. In the deterministic simulation of the full model under seasonality, colonies with *l*_0_≤300 are always infected (for example, see Fig 6A and 6B), while colonies with *l*_0_>300 are always uninfected (for example, see Fig 6C and 6D). In stochastic simulations (Fig 8B, black line), however, the percentage of colony survival is found to decrease as the rate of egg-laying initially increases (*l*_0_ from 50 to 200), but later increase as the laying rate becomes higher (*l*_0_>200). As the rate of egg-laying initially increases, the population size also increases, which associates with a higher probability of disease transmission due to a higher frequency of interactions between individuals. Therefore, from *l*_0_ equal to 50 to 200, the survival percentage gradually drops. In previous sections, we have shown that larger colonies are more effective in performing the hygienic response. Therefore, as the laying rate becomes very high (*l*_0_>200), the rate at which infected bees are removed gradually exceeds the rate of transmission; thus, the survival is more observed. Our results again confirm the role of hygienic behavior in reducing disease spread in large-size honeybee colonies.

Next, we investigate how social segregation in our model contributes to the social immunity of the colony. When the hygienic behavior is removed (*k*_rem_ = 0), the colonies in Fig 8B, red line, experience a similar decrease in the chance of colony survival as the rate of egg-laying initially increases, but as the laying rate becomes very high, the chance of survival remains at a minimum value around 70%. An initial decrease in the colony survival as the laying rate increases is due to a higher frequency of interactions between individuals, which facilitates disease transmission, as described above. However, what makes the percentage of survived colonies remain at 70%, rather than continually decrease as the laying rate becomes higher, is the colony’s social segregation. Foragers only interact with nectar-receivers, but not with other hive bees (e.g., nurses). Infected foragers that successfully unload nectar need to go for another round of nectar-collecting before they can transmit the disease again. Therefore, with the severely impaired homing ability demonstrated here (*p*_surv_ = 0), an infected forager has only one chance with a probability equal to *p*_t2_ (= 0.3 in the simulation) to spread the disease into the nest. Increasing *p*_surv_ to 0.5 (Fig 8B, blue line) decreases the minimum percentage of survived colonies to around 60%, which again remains at the value even when the rate of egg-laying becomes very high.

We then compare simulations of colonies exhibiting social segregation (Fig 8B, red and blue lines) to another simulation setting, where we suppress all social immunity mechanisms in the model (Fig 8B, purple line). 1) we set *k*_rem_ = 0 to remove the hygienic behavior. 2) we set *p*_surv_ = 0.5 to elevate homing ability of infected foragers. 3) one infected nectar-receiver (iR_1_) is introduced instead of an infected forager in early spring to bypass the social segregation mechanism between foragers and nurses. As a result, the percentage of survived colonies drops to almost 0% as the rate of egg-laying becomes very high (Fig 8B, purple line).

Fig 8C explores two more cases in which social segregation is compromised (one infected nectar-receiver (iR_1_) is introduced in early spring and *p*_surv_ = 0.0 or 0.5), but with intact hygienic behavior (*k*_rem_ = 2.5 × 10^−3^). The plot shows that without the social segregation mechanism, colonies suffer severe losses as the laying rate initially increases. However, colonies with higher laying rates can effectively mitigate the losses. Therefore, it becomes evident from our simulations that organizational immunity in terms of social segregation combined with impaired homing ability of infected foragers forms the first line of defense to reduce the uptake of pathogens into the colony. If this first line of defense fails, the hygienic behavior (*k*_rem_>0) provides another mechanism in the prevention of disease spread within the colony.

Finally, we investigate in Fig 8D the effect of the onset of an infection in relation to the timing of spring. Here, we introduce one infected forager returning from nectar-collecting at day *t*_0_ after the onset of spring (i.e., iF_1_(*t*_0_) = 1). It is shown that colonies are most vulnerable during a couple first weeks of spring. Colonies exposed to an infection earlier in spring, when the colony’s population size is still small, experience higher disease transmission risk. Colonies exposed to an infection in late spring, when the colony’s population has already risen, can prevent or reduce the disease risk.

Infection in simulations in Fig 8 is introduced as one infected forager returning from nectar-collecting (iF_1_) at the onset of spring. S1 Fig plots simulation results when an infection is introduced constantly over non-winter months. In the simulations, returning foragers have a probability *p*_inf_ of becoming infected. The plot shows that the results remain in qualitative agreement with those in Fig 8, although quantitative differences can be seen.

## Conclusion

Trophallaxis, which is also observed in ants, termites, and wasps, has been evolved as cooperative and mutualistic relationships between members in highly social groups [48, 67]. As trophallaxis is essential for communication and nectar distribution in honeybee colonies, it may play a critical role in disease transmission. We developed a generic model describing how an infection spreads within the colony when bee members engage in the trophallactic activity to distribute nectar. One possible application of our model is to explain the transmission of viruses within the colonies. Detection of viruses in hypopharyngeal glands of infected workers [16–18] implies trophallactic feeding as a transmission pathway from nurses to brood and among worker bees.

Many honeybee viruses have been documented to be involved in colony mortality. A metagenomic study revealed that IAPV is strongly correlated with CCD-affected colonies in the US [68]. Chen et al. [16] reported a negative correlation between the level of IAPV infections and the size of infected colonies, which is also negatively associated with the overwintering mortality of the colonies. The presence of DWV and ABPV is significantly related to winter losses of colonies in Switzerland [69] and Germany [70]. These viruses can persist in all developmental stages of honeybees at low levels without a noticeable impact on the individual and colony health. However, the viral infection accompanied by a high varroa infestation level can cause the viruses to become more virulent [56, 71–73]. *V. destructor* is necessary as a vector to overtly transmit DWV to pupae, causing pupal death and the emergence of inviable adult bees with a clinical symptom of deformed wings [73]. Our current model, however, did not include the mites. In fact, viral infection can impact on colony survival while the infection remains asymptomatic [16]. Our study demonstrated how subclinical infection could lead to colony losses in weak colonies (e.g., colonies with low laying rates). Nevertheless, explicitly adding mites and their role as a transmission vector into our future model will generate richer transmission dynamics as shown in previously published models [34–36, 74]. Subclinical infection, as simulated in our model, is also assumed to rule out the fecal-oral transmission as honeybees in healthy colonies normally defecate outside the hive.

In social insects, it is known that increasing the population size associates with a higher probability of disease transmission within the colonies due to a higher frequency of interactions between individuals [24]. Here, we showed that two strategies utilized by honeybees could reduce the risk of disease spread at the colony level, especially in large-size colonies. The first strategy is the organization of the interaction pattern that limits the infection risk of valuable individuals (nurses and brood) from high-risk individuals (foragers), known as ‘organizational immunity.’ Unlike previous models [37–39, 41, 42] that considered all hive-bees together as one entity, our model explicitly separated nurses and nectar-receivers into two different classes. As a result of the social and spatial segregation, we assume that disease transmits sequentially from foragers, nectar-receivers, nurses, to brood. Infected foragers cannot spread infection directly to nurses or brood. In addition, infected foragers (e.g., IAPV-infected) have an impaired homing ability compared to healthy foragers [60]. Therefore, the infected foragers can transmit pathogens to nectar-receivers only if they make a successful return from nectar-collecting. We demonstrated with stochastic simulations that the social segregation, combined with the impaired homing ability of infected foragers, limits the risk of pathogen uptake into the colony below a certain level.

The second strategy involves the hygienic behavior performed by nurse bees in detecting and removing infected bees (brood and workers). Our model that incorporated the behavior showed the colony-level defense against disease spread within the colonies. More importantly, we showed that the egg-laying rate influences the hygienic behavior. Larger colonies tolerate infectious pathogens better than smaller colonies. We propose that the hygienic behavior provides a crucial feature that protects horizontal disease transmission among honeybees who reside inside the nest and acts as a counter-mechanism against pathogens’ exploitation of frequent interactions in large colonies. Our finding is in concert with Chen et al. [16], who observed that large colonies suffer lower IAPV infection levels than small colonies.

Reducing colony size, specifically, the nurse population, weakens the colony’s hygienic response and potentially results in colony losses. We demonstrated that seasonality exerts a significant effect on the colony status. A reduction in the bee population in winter leaves colonies in a vulnerable state as the hygienic response mechanism is impaired. When the nectar flow begins in the spring, active nectar-distributing can enhance rapid pathogen spread within the colony, leading to colony failure. This could potentially explain frequent and widespread observations of sudden colony losses in early spring (the “spring dwindle”) [3]. It also implies that small colonies, such as managed colonies, are likely to experience a sudden death in early spring in the face of an infection. This finding is in line with a study observing that all large colonies infected with IAPV survived through the winter months, while almost all small colonies infected with IAPV died before spring [16].

Additionally, as the egg-laying capacity declines gradually as the queens age, we predict that colonies headed by old queens may have a lower chance of overwintering survival. Consistently, the queen age was identified among multiple other factors, including mite infestation, DWV infection, and ABPV infection, to contribute to winter colony losses in Germany [70].

The role of a critical colony size that maintains the colony growth and survival was proposed in other work before. For example, several studies [36–38, 41, 42, 75] proposed that a certain number of the worker population is critical to maintain the brood-rearing activity, which is expressed as an eclosion rate. The colony mortality is ensured when the rate of honeybee death exceeds the rate of successful rearing. Our model did not incorporate such a mechanism as we assume a constant eclosion rate. Instead, our study provided evidence for colony size as a critical factor in maintaining the hygienic response as part of the social immunity against disease transmission within the colonies.

Our model can be extended in several ways. Social processes and regulations such as social inhibition (foragers slow down hive-to-forager development) and cooperative brood rearing by worker bees were ignored in the present model. These processes are of important factors determining the colony’s fate, as proposed in [37–39]. These processes can be incorporated into the future version of our model to explore their interplay with the social immunity mechanisms. Also, behavioral changes of individual bees upon infection could be examined. For example, IAPV-infected bees were observed to engage less in trophallaxis with other nest-mates, presumably, to reduce pathogen transmission [76].

In summary, the present work studied two social immunity features of honeybee colonies. We showed that an organizational interaction pattern that excludes or limits foragers from interacting with bees residing in the inner parts of the nest forms the first line of defense to reduce the uptake of pathogens into the colony. However, if honeybees in the inner parts of the nest become infectious, the hygienic behavior of nurse bees toward sick bees acts as another defense line to reduce the disease risk at the colony level. Despite these collectively performed defenses of colony members, our model suggested that winter conditions that reduce colony population size combined with an infection can compromise the social immunity defenses and drive the colony to mortality. We also emphasized the role of stochasticity in the fluctuation of the bee population that drives two colonies under identical conditions into different fates, an observation that cannot be predicted by the deterministic model.

## Supporting information

S1 Text Parameter estimation. (PDF)

S2 Text Stochastic model. (PDF)

S1 Fig Stochastic model with a constant probability of infection. The figure compares the model with an infection proposed in the main text (one infected forager is introduced at the onset of infection, iF_1_(0) = 1) (left panel) with the model that allows returning foragers to be infected bees (F_0_ → iF_1_) with a probability *p*_inf_ (right panel). The two models are in qualitative agreement although quantitative differences can be seen.
(PDF)

S1 File Core model in XPP format. (ODE)

S2 File Full model in XPP format. (ODE)

S3 File Stochastic model in C++ format. (CPP)
