# Supplementary material for: A model of infection in honeybee colonies with social immunity
Source: PLoS One. 2021 Feb 22;16(2):e0247294. doi: 10.1371/journal.pone.0247294 (PMC7899363; doi:10.1371/journal.pone.0247294)
Supplement: S1 Text — (PDF) [file pone.0247294.s001.pdf]

PLoS One S1 Text

Article title: A Model of Infection in Honeybee Colonies with Social Immunity

Authors: Laomettachit, T., Liangruksa, M., Termsaithong T., Tangthanawatsakul A., Duangphakdee, O.

Parameter estimation

Contact rate between nurse bees and brood (k_{NB})
Heimken et al. (2009) [1] observed two feeding visits per 30 min (0.06 visit/min) by nurse bees per larva in an experimental colony of 2000–3000 workers. Assuming the colony has around 700 nurse bees, we estimate k_{NB} in our model to be 0.06/700 contacts/bee/min or ~0.1 contacts/bee/day, so that k_{NB}xN results in two nurse contacts per 30 min per larva.

Contact rate between nectar-receiver and nurse bees (k_{RN})
Each nectar-receiver makes around 0.4–1 trophallactic contacts per min [2], 60% of which are with nurses [3]. Therefore, we assume 0.7x0.6=0.42 nurse contacts/min/nectar-receiver. The observation was made in an experimental colony of around 4000 workers [2]. Assuming the colony has around 1200 nurse bees, we estimate k_{RN} in our model to be 0.42/1200=0.00035 contacts/bee/min or ~0.5 contacts/bee/day so that k_{RN}xN results in 0.42 nurse contacts/min/nectar-receiver.

Contact rate between foragers and nectar-receivers (k_{FR})
De Marco and Farina (2001) [4] observed that each forager performs one to two effective food transfers (trophallaxes) per hive stay to nectar-receivers. We assume that the forager spends, on average, 100 s (1.6 min) to successfully search for and unload the nectar to a nectar-receiver. Therefore, the rate of searching and unloading is 1/1.6=0.625 per min per forager. The observation was made in an experimental colony of nearly 2000 workers [4]. Assuming the colony has around 600 nectar-receivers, we estimate k_{FR} in our model to be 0.625/600=0.001 contacts/bee/min or ~1.44 contacts/bee/day, so that k_{FR}xR results in 0.625 nectar-receiver contacts/min/forager.

References

[1] Heimken C, Aumeier P, Kirchner WH. Mechanisms of food provisioning of honeybee larvae by worker bees. J Exp Biol. 2009;212(Pt 7):1032-5. doi: 10.1242/jeb.022582. PubMed PMID: 19282500.

[2] Seeley TD. Social foraging in honey bees: How nectar foragers assess their colony's nutritional status. Behav Ecol Sociobiol. 1989;24(3):181-99. doi: 10.1007/BF00292101.

[3] Grüter C, Farina WM. Nectar distribution and its relation to food quality in honeybee (*Apis mellifera*) colonies. Insectes Soc. 2007;54(1):87-94. doi: 10.1007/s00040-007-0915-z.

[4] De Marco R, Farina W. Changes in food source profitability affect the trophallactic and dance behavior of forager honeybees (*Apis mellifera* L.). Behav Ecol Sociobiol. 2001;50(5):441-9. doi: 10.1007/s002650100382.
