# Supplementary material for: A model of infection in honeybee colonies with social immunity
Source: PLoS One. 2021 Feb 22;16(2):e0247294. doi: 10.1371/journal.pone.0247294 (PMC7899363; doi:10.1371/journal.pone.0247294)
Supplement: S2 Text — (PDF) [file pone.0247294.s002.pdf]

PLoS One S2 Text

Article title: A Model of Infection in Honeybee Colonies with Social Immunity

Authors: Laomettachit, T., Liangruksa, M., Termsaithong T., Tangthanawatsakul A., Duangphakdee, O.

Stochastic model

Our stochastic model is implemented by the Gillespie's stochastic simulation algorithm (SSA) [1], which is the most accurate way to simulate chemical reaction systems numerically. We have applied the approach to study stochastic fluctuations in honeybee colonies before [2,3]. In the stochastic model, each term from the deterministic model is considered as a stochastic event. There are 25 possible events in the model (Table S1), and in the infinitesimal time interval, an event occurs with a probability proportional to the propensity rate.

Iteratively, the model determines the time interval (τ) until the next event occurs, and the index of the next event to occur. Let $a_0=\sum a_i$, where i is the event index, and a_i is the propensity function for event i. Then, $\tau = -\ln(r_1)/a_0$ and the next event to occur is determined by the smallest integer index i that satisfies $\sum_{i'=1}^{i} a_{i'} > r_2 a_0$, where r_1 and r_2 are two independent random numbers drawn from the uniform (0,1) distribution. Once an event index is selected, the state transition corresponding to the event index in Table S1 is applied, and the simulation time is updated by adding the current time by τ. This process will iterate until the simulation reaches a specified end time.

Table S1 The propensity rates and the state transitions of the stochastic model

Event index (i)	Propensity rate (a_i)	State transition	Event definition
1	$k_r \cdot F_0$	$F_0 \rightarrow F_1$	An empty forager leaves the nest to forage, and returns as a nectar-loaded forager.
2	$k_r \cdot iF_0$	If $r_3 \leq p_{surv}$, $iF_0 \rightarrow iF_1$, else $iF_0 \rightarrow \phi$	An infected empty forager leaves the nest to forage, and returns as an infected nectar-loaded forager with a probability p_{surv} or disappears in the field with a probability $(1-p_{surv})$.
3	$k_{FR} \cdot F_1 \cdot R_0$	$F_1 \rightarrow F_0$, $R_0 \rightarrow R_1$	A loaded forager unloads nectar to an empty nectar-receiver.
4	$k_{FR} \cdot F_1 \cdot iR_0$	$F_1 \rightarrow F_0$, $iR_0 \rightarrow iR_1$	A loaded forager unloads nectar to an infected empty nectar-receiver.
5	$k_{FR} \cdot iF_1 \cdot R_0$	$iF_1 \rightarrow iF_0$,<br>If $r_3 \leq p_{t2}$, $R_0 \rightarrow iR_1$,<br>else $R_0 \rightarrow R_1$	An infected loaded forager unloads nectar to an empty nectar-receiver. The latter becomes infected with a probability p_{t2}.
6	$k_{FR} \cdot iF_1 \cdot iR_0$	$iF_1 \rightarrow iF_0$, $iR_0 \rightarrow iR_1$	An infected loaded forager unloads nectar to an infected empty nectar-receiver.
7	R_1/t_s	$R_1 \rightarrow R_0$	A loaded nectar-receiver deposits nectar in honey cells and returns to the delivery area.
8	iR_1/t_s	$iR_1 \rightarrow iR_0$	An infected loaded nectar-receiver deposits nectar in honey cells and returns to the delivery area.
9	$p_{t1} \cdot k_{RN} \cdot iR_1 \cdot N$	$N \rightarrow iN$	A nurse becomes infected when contacting an infected nectar-receiver.
10	$p_{t0} \cdot k_{NB} \cdot iN \cdot B$	$B \rightarrow iB$	A brood becomes infected when contacting an infected nurse.
11	$k_{rem} \cdot N \cdot iN$	$iN \rightarrow \phi$	An infected nurse is removed by a healthy nurse.
12	$k_{rem} \cdot N \cdot iB$	$iB \rightarrow \phi$	An infected brood is removed by a healthy nurse.
13	l_0	$\rightarrow B$	Birth of a new brood
14	B/n_B	$B \rightarrow N$	A brood develops to a nurse.
15	iB/n_B	$iB \rightarrow iN$	An infected brood develops to an infected nurse.
16	N/n_N	$N \rightarrow R_0$	A nurse develops to a nectar-receiver.

Event index (i)	Propensity rate (a_i)	State transition	Event definition
17	iN/n_N	$iN \rightarrow iR_0$	An infected nurse develops to an infected nectar-receiver.
18	R_0/n_R	$R_0 \rightarrow F_0$	A nectar-receiver develops to a forager.
19	iR_0/n_R	$iR_0 \rightarrow iF_0$	An infected nectar-receiver develops to an infected forager.
20	R_1/n_R	$R_1 \rightarrow F_1$	A nectar-receiver develops to a forager.
21	iR_1/n_R	$iR_1 \rightarrow iF_1$	An infected nectar-receiver develops to an infected forager.
22	F_0/n_F	$F_0 \rightarrow \phi$	A forager dies.
23	iF_0/n_F	$iF_0 \rightarrow \phi$	An infected forager dies.
24	F_1/n_F	$F_1 \rightarrow \phi$	A forager dies.
25	iF_1/n_F	$iF_1 \rightarrow \phi$	An infected forager dies.

ϕ represents 'death'.
r_3 is a random number drawn from the uniform (0,1) distribution.

References

[1] Gillespie DT. Stochastic simulation of chemical kinetics. Annu Rev Phys Chem. 2007;58:35-55.
[2] Laomettachit T, Termsaithong T, Sae-Tang A, Duangphakdee O. Decision-making in honeybee swarms based on quality and distance information of candidate nest sites. J. Theor. Biol. 2015;364:21-30. doi: https://doi.org/10.1016/j.jtbi.2014.09.005.
[3] Laomettachit T, Termsaithong T, Sae-Tang A, Duangphakdee O. Stop-signaling reduces split decisions without impairing accuracy in the honeybee nest-site selection process. J Insect Behav. 2016;29(5):557-77. doi: 10.1007/s10905-016-9581-1.
