# Supplementary figures and images for: A model of infection in honeybee colonies with social immunity
Source: PLoS One. 2021 Feb 22;16(2):e0247294. doi: 10.1371/journal.pone.0247294 (PMC7899363; doi:10.1371/journal.pone.0247294)

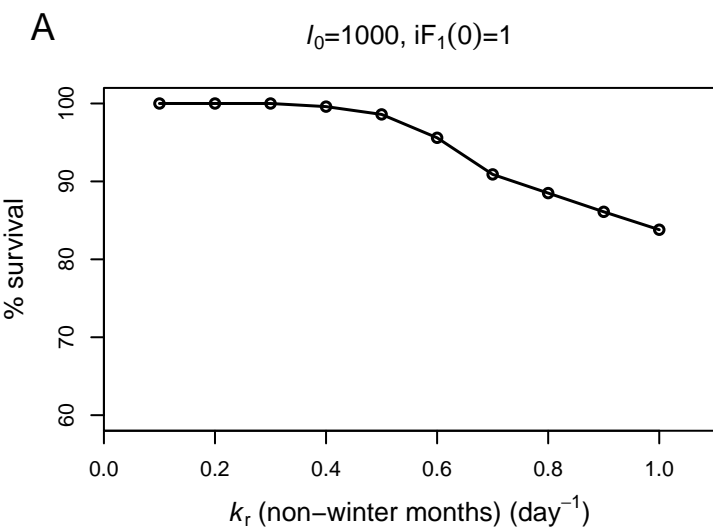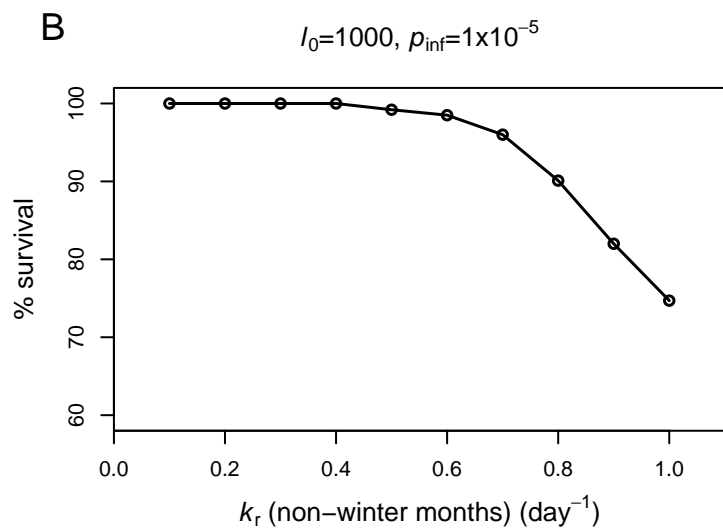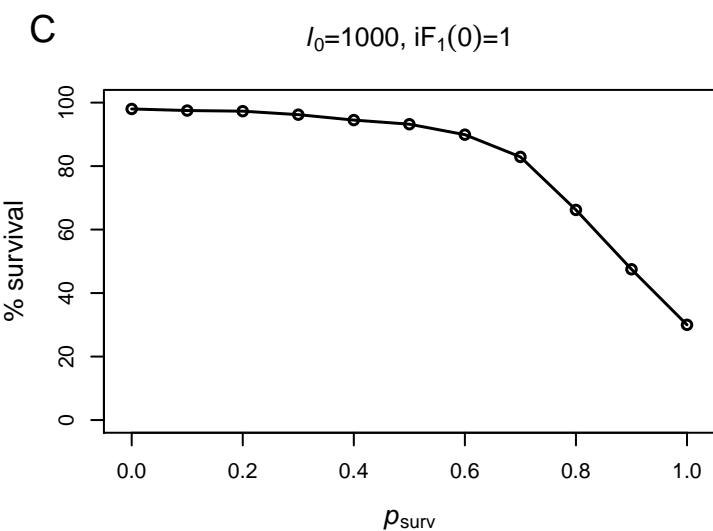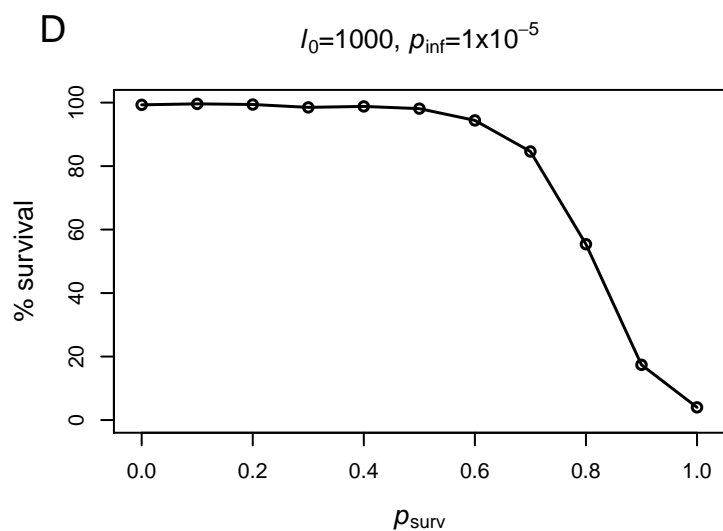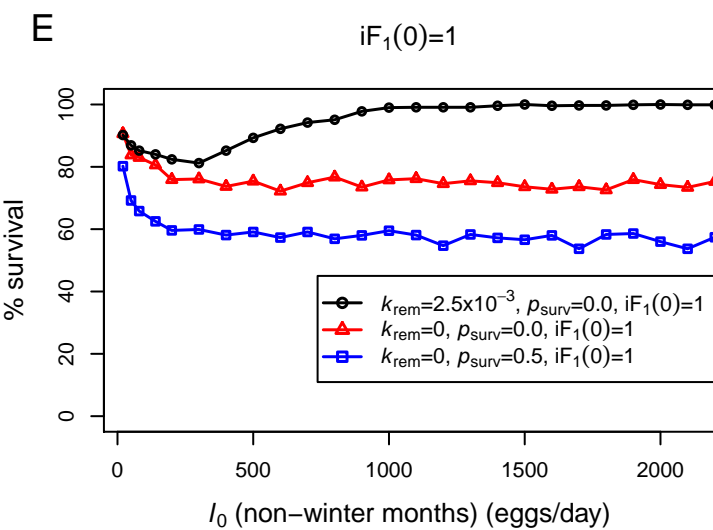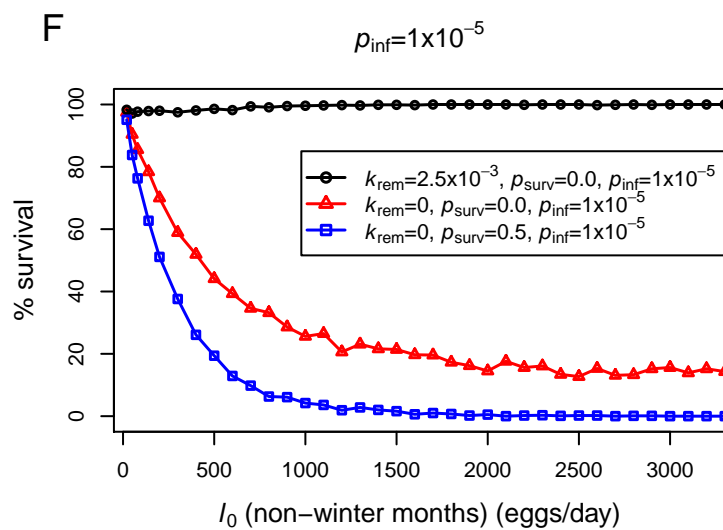

Supplement: S1 Fig — The figure compares the model with an infection proposed in the main text (one infected forager is introduced at the onset of infection, iF1(0) = 1) (left panel) with the model that allows returning foragers to be infected bees (F0 → iF1) with a probability pinf (right panel). The two models are in qualitative agreement although quantitative differences can be seen. (PDF) [file pone.0247294.s003.pdf]
