# Supplementary material for: A model of infection in honeybee colonies with social immunity
Source: PLoS One. 2021 Feb 22;16(2):e0247294. doi: 10.1371/journal.pone.0247294 (PMC7899363; doi:10.1371/journal.pone.0247294)
Supplement: Attachment — Submitted filename: PONE_manuscript_082920_report.pdf [file pone.0247294.s007.pdf]

Review of *A Model of Nosema ceranae Infection in Honeybee Colonies with Social Immunity*, T. Laomettachit et al.

Overview: This paper develops and analyzes a new deterministic differential equation model, along with a related stochastic model, for *Nosema ceranae* disease dynamics within a honeybee colony. The model divides the colony into four life stages (brood, nurses, nectar-receivers, and foragers), which effectively creates a socially segregated chain of disease transmission. The model also assumes that honeybees engage in a hygienic response by removing infected bees. Numerical experiments show that the social segregation and hygienic response, as well as colony size and seasonality, influence the risk of colony collapse.

General Comments: The authors present a relatively simple model of within-hive disease dynamics that elucidates the role of social immunity and hygienic behavior on honeybee colony survival or collapse. The assumptions and model parameters are rooted carefully in the biological literature, and the analysis is straightforward, thorough, and intuitively presented. Most of the results are expected, but some results are surprising (e.g., non-monotonic relationship between laying rate and colony collapse frequency). The manuscript is both succinct and thorough; it is effortless to understand and a pleasure to read.

I only have two major questions that need to be addressed before I recommend the manuscript be accepted. All other comments are minor, but addressing them would improve the manuscript.

Major Comments:

- ☐ Extended model: Why are there no new forager infections (i.e., no transitions $F_0 \to iF_1$)? I suspect that the assumption of only one infected forager returning to the hive affects many of the presented results.

- ☐ pg 11, ln 360-371: The case $iR_1(0) = 1$ is only explored in an extreme circumstance (hygienic behavior and impaired homing both failed). What would happen if social segregation and impaired homing failed, but hygienic behavior remained ($k_{rem} = 2.5e-3, p_{surv} = 0.5$ and $iR_1(0) = 1$)? What would happen if social segregation failed, but impaired homing and hygienic behavior remained ($k_{rem} = 2.5e-3, p_{surv} = 0$ and $iR_1(0) = 1$)? I would trust the conclusions more if these cases were explored.

Minor Comments:

- ☐ pg 2, ln 27-29: What is the biological mechanism responsible for reduced lifespan, reduced colony size, reduced honey production, and increased mortality? Knowing the mechanism will help the reader assess the plausibility of model assumptions.

- ☐ pg 4, ln 115-117: What is the biological mechanism for infection detection and removal? Knowing this will also help the reader assess the plausibility of model assumptions.

- ☐ Core model: Is there biological evidence for maturation while infected without any significant disease mortality? Disease mortality is mentioned much later on pg 8, ln 250-253, and I think this should be moved into the core model section.

- ☐ pg 5, ln 132-133: Please cite the references from Table 1 when claiming $n_B = 20$ and $n_N = 10$.

- ☐ Table 1: It is assumed that transmission probabilities given contact are identical for the brood, nurses and nectar-receivers. Is there biological evidence for this assumption, or is it made for simplicity?

- ☐ pg 5, ln 145: Is there no chance a nurse will get infected in the removal process? I imagine that killing an infected bee might risk transmission. The model also assumes that infected nurses would not attempt to remove infected brood or workers. Is this know, and if so, what is the biological mechanism preventing infected nurses from enacting hygienic behavior?
- ☐ pg 5, ln 161-162: In the core model, $p_{t1}k_{RN}iR_1 = 5e - 4$. Why is this selection made?
- ☐ Figure 2: How are these figures generated (analytical or numerical)?
- ☐ Figure 3: Laying rate is used as a proxy for colony size, and while a larger laying rate will under most circumstances lead to larger colony size, I find it a bit misleading to use laying rate and colony size (or brood size or nurse numbers) nearly interchangeably.
- ☐ Figure 3: A bifurcation diagram of $B(0)$ versus $N(0)$ may be informative in the bistable region. I suspect the ratio of brood to nurses is important.
- ☐ Figure 3: In subfigure B, consider removing the cross and asterisk markers in favor of an arrow or letters. Asterisks imply a bifurcation point in MatCont, and I found it confusing.
- ☐ pg 8, ln 227-228: Please cite the references from Table 1 when claiming $n_R = 11$ and $n_F = 14$.
- ☐ pg 9, ln 287-289: Would model results drastically change if the laying rate were explicitly dependent on foraging?
- ☐ pg 9-10, ln 295-298: Is there biological evidence that larger colonies are less likely to collapse due to disease?
- ☐ pg 10, ln 316: What is the logic behind the 0.8 infection threshold? Is there biological evidence that a hive will collapse once 80% of the colony is infected, or was this an arbitrary threshold?
- ☐ pg 11, ln 337-338: Please explain the intuition behind the impaired hygienic response for moderate laying rates. I did not understand precisely which model assumption(s) led to this result.
- ☐ Figure 7: These figures would be easier to digest if the y-axis were % survival rather than % collapse. All previous figures had survival on the y-axis.
- ☐ Starting in the Results section, the manuscript contains several grammatical errors and typos. None impair understanding except on pg 12, ln 405.
- ☐ pg 13, ln 465-475: For the sake of flow, consider moving this paragraph before the "In summary..." paragraph.
