# Supplementary material for: A model of infection in honeybee colonies with social immunity
Source: PLoS One. 2021 Feb 22;16(2):e0247294. doi: 10.1371/journal.pone.0247294 (PMC7899363; doi:10.1371/journal.pone.0247294)
Supplement: Attachment — Submitted filename: Review Comment PONE-D-20-25709.pdf [file pone.0247294.s008.pdf]

Review – PONE-D-20-25709 – A Model of Nosema ceranae Infection in Honeybee Colonies with Social Immunity

Summary of Comments

Main Comments

This is one of many technically good mathematical models attempting to understand the dynamics and infection processes underpinning honey bee colony loss. Whilst the authors' capturing of interaction structure and social hygienic behaviours is of interest, worthy of pursuit, and will contribute to the field, the current paper sits an awkward place of alleging to be pathogen specific without doing an adequate job of capturing that pathogen's biology (see some points below). However, the presentation of the manuscript lacks generality or reference to which other pathogens it might represent, and where it sits comparatively with other demographic or epidemiological models. I have given some discussion below as to my objections to some of the core biology underpinning the model, which will need revision.

I do believe this work will be useful, but I recommend the authors commit to either: 1) a Nosema ceranae specific model with much greater exploration of the true biology of the pathogen, including its density-dependant airborne transmission and facultative precocial foraging by bees upon infection; or 2) a much more generic model where they explore parameter space more generally, remove their claims to this being a specific study of the Nosema system, and more generally overview which pathogens exhibit biology close to what they are modelling. I also believe they should better explicitly reference what their model does similarly and differently to other models in this system, some of which they reference well, others poorly, and some are missed.

Introduction

The introduction is long for the paper and will likely require some changes following my recommendations below.
In particular, Lines 1-21: This section reads as extremely generic and, in places, it is incorrect. For example, 'CCD' has been well hypothesised as a symptom of elevated forager mortality following honey bee modelling work by Khoury et al 2011, work which the authors do cite later in the introduction but without this clarity. Much of the reference to CCD, which is no longer considered a useful term by honey bee biologists, seems to be done as a generic replacement for colony death (precisely why we now discourage its use). The authors state that worldwide, colonies are declining (Line 5). This is simply incorrect (see UN FAO data showing worldwide increase in honey bee colonies despite declines in parts of NW Europe and North America).

I also believe the authors miss some critical studies honey bee mathematical modelling which their work should be framed against, for example Booton et al 2017 https://www.sciencedirect.com/science/article/abs/pii/S0022519317301212 and Bartlett et al and Bartlett et al 2019 https://besjournals.onlinelibrary.wiley.com/doi/full/10.1111/1365-2664.13461 both of which include aspects of this work which are, to my view, missed. For theory to remain cohesive and integrated within the field of honey bee biology, more explicit reference must be made to similarities and differences between model studies. I would ask the authors to explicitly state in the introduction which parts of other models they capture, which they do not, and what they introduce which is new in the context of honey bee mathematical modelling.

Methods, Results, Discussion

The results and discussion are well presented and seem to my reading correct in their derivations. However, I do not believe the core biological assumptions made can be justified. For example, the authors themselves state that there is no evidence of the removal of Nosema infected brood, but include it regardless. I do not object to the modelling in principle, but the study should not be presented as a study of a specific disease system if it does not reflect the known biology. On this topic, it has been experimentally shown that Nosema infections can occur without trophallaxis (a frequency-dependant infection dynamic) simply via close, non-contact proximity (and that in this regard, it is a density-dependant infection dynamic). Roberts & Hughes 2015 (https://www.cambridge.org/core/journals/parasitology/article/horizontal-transmission-of-a-parasite-is-influenced-by-infected-host-phenotype-and-density/B05BBB47CE18A0A7F8323489F2C322FC) showed this specifically for *Nosema ceranae* in honeybees. Given this, I do not believe the model suitable for the specific studying of Nosema infections in honey bee colonies. If the authors wish to remain specifically studying Nosema, I believe the model must be revised. If they wish to present this as a generic model including both infected culling and infection purely via direct interaction then they must justify this biologically and remove explicit claims of modelling Nosema. This extends to their citing of Goblirsch et al for the additional mortality at the foraging stage of Nosema infection. Goblirsch et al explicitly state that infection with Nosema induces preferential abandonment of in-colony duties and a precocial foraging, which would have serious implications for their models behaviour and the conclusions to be drawn from it. I realise the authors mention this extension in line 473-475, but I believe the importance of this point to justifies its inclusion in the original model.

I do however believe the model framework in how it addresses hygienic behaviour & social culling, forager/ receiver / onward transmission, and the implications thereof to be strong and worth publishing with modification, although I do also believe that a flexible task allocation and infection-dependant precocial ageing into the forager class should be included in this (see above reference and many others).

I have not given detailed comments beyond this, as I consider these biological problems with the model's mandate to require enough revision that additional commentary on more detailed aspects of the work to not remain relevant. However, I would be happy grateful to review this manuscript again in more detail if the authors agree with some of these recommendations.

Conclusion

Similarly, I expect part of the conclusion to change if the biological rationale is improved. I also mirror my comments regarding the introduction that the conclusion is very generic in its discussion around compound stressors and alleged 'CCD', for example Lines 433-445 have little relevance to the work here as they are currently written. If the authors wish to discuss a variety of pathogens of honeybees, it should be done with regards to their known biology, and whether the interaction structure and social hygienic behaviours they present would behave similarly or differently. It is critical the authors make it clear what parasites they believe these findings to apply to, and which they do not. Note I do not believe this model currently approximates Nosema biology well enough to be stated as a study of Nosema ceranae infection dynamics.
