# Supplementary material for: A model of infection in honeybee colonies with social immunity
Source: PLoS One. 2021 Feb 22;16(2):e0247294. doi: 10.1371/journal.pone.0247294 (PMC7899363; doi:10.1371/journal.pone.0247294)
Supplement: Attachment — Submitted filename: Response to Reviewers.pdf [file pone.0247294.s009.pdf]

Authors' responses to editor and reviewers
PONE-D-20-25709 A Model of *Nosema ceranae* Infection in Honeybee Colonies with Social Immunity

Editor Comments

1) As you can see from the reviews below, each reviewer thought this manuscript has the potential to provide a valuable contribution to the field and has many positive aspects. However, each of the reviewers also brought up important points that will need addressing or revision.
**Authors' response:**<br>Thank you for giving us the opportunity to resubmit the manuscript for a possible publication in PLOS ONE. We are grateful for the time and effort that you and the reviewers dedicated to provide feedbacks and insightful comments for improving our paper. Below we provide point-by-point responses to the reviewers' comments.
2) In particular, there are several issues with assumptions based on the known biology of honey bees and Nosema infection, many of which are pointed out by the reviewers. I recommend the authors carefully address these points in the revision. I feel that the reviewers have provided extremely helpful advice, with references, to assist the authors in these revisions.
**Authors' response:**<br>In the revised manuscript, we have followed the recommendation by Reviewer #3 to present our model as a more general model to a communicable disease transmitted through trophallaxis as you will see in our responses to the reviewers below. We also change the title of our manuscript to 'A Model of Infection in Honeybee Colonies with Social Immunity'.
3) I will also highlight Reviewer #3's note about the use of "CCD" as a blanket for all colony deaths/bee losses. This term has a very specific definition that quickly became diluted by misuse and is thus only used in current honey bee research literature under very specific conditions. They also highlight the care needed in noting 'bee declines' as they are correct in their point that colony numbers are not declining in the last decade (some areas, like the USA, have seen declines in numbers since the mid-20th century, but these changes are due to complex factors). Instead, I recommend the authors look into data on colony losses as a component of beekeeping costs; i.e., beekeepers require greater monetary input to keep colony numbers at the needed level.
**Authors' response:**<br>We now avoid using "CCD" as a blanket for all colony losses in our revised manuscript. We also revise our statement about 'bee declines' to also present the fact that the global number of managed colonies has risen. We also point out that 'honey bee management has been challenged with increased pathogen and environmental pressure associating with increased beekeeping cost' as suggested.

Reviewer #1 Comments

1) The authors propose a model for honey bee colony collapse that originates when foragers infected with *Nosema ceranae* engage in trophallactic exchange with uninfected nectar-receivers. Their model is based on a series of parameters (e.g., egg-laying rate, duration of caste-specific roles, forager homing probability) whose input into deterministic and stochastic simulations establishes scenarios under which a colony may fail from Nosema disease. Most parameter values are supported by the literature. The model also considers the role of social segregation and hygienic behavior in creating first and second lines of defense against the spread of *N. ceranae* from forager to nectar-receiver to nurse bee to brood; this sequence of contact is important to the model. The take home message is that a large colony with a strong nurse bee population can buffer a high level of infection as long as the rate of egg-laying surpasses the rate of hygienic removal of infected individuals. In stochastic simulations, it is not only colony size but the failure of infected foragers to return to the colony coupled to social segregation (i.e., lack of contact of uninfected nectar-receivers with infected foragers) that will increase chances of colony survival.

Of concern is that the model relies heavily on two assumptions that have not received much attention in our understanding of Nosema disease: oral-oral transmission and infection of immature stages. A widely-accepted route of horizontal transmission of Nosema in honey bees is fecal-oral where bees ingest spores from contaminated feces. Moreover, acquisition of spores by this route may not necessarily be restricted to foragers outside the hive, as is assumed by the model. Oral-oral transmission has been tested under laboratory conditions using caged bees (Smith, 2012); however, there has been little additional work in this area. A justification for why oral-oral transmission was the preferred route should be made more apparent.

Authors' response:

We would like to express our gratitude for your insightful comments.

We remove the claims about our model being a specific case for *Nosema ceranae*. Instead, we now present our work as a more general model to a communicable disease transmitted through trophallaxis with a possible application to explain viral transmission within the colonies. We also change the title of our manuscript to 'A Model of Infection in Honeybee Colonies with Social Immunity'.

To justify why oral-oral transmission is the preferred route, we add a new paragraph discussing the role of trophallaxis in viral transmission.

Line 20–31:

Honeybees living in large colonies are prone to the rapid spreading of pathogens among individuals due to high population density and high contact rates. Trophallaxis (mouth-to-mouth food sharing) is considered a routine behavior that facilitates pathogen transmission [12, 13]. For example, trophallaxis may be the predominant mechanism of horizontal viral transmission. Nurse bees infected with viruses can transmit them to the larvae via trophallaxis since they rely heavily on nurses' tending and feeding. The transmission route is supported by the detection of viruses, such as deformed wing virus (DWV) [14], sacbrood virus (SBV) [15], and Israeli acute paralysis virus (IAPV) [16], in larval food. In addition, the detection of viruses in the hypopharyngeal gland of infected worker bees was demonstrated for acute bee paralysis virus (ABPV) [17], sacbrood virus [18], and Israeli acute paralysis virus (IAPV) [16], implying a possible foodborne transmission route driven by trophallaxis.

In the Discussion section, we point out a possible application of our model to explain viral transmission.

Line 418–444:

We developed a generic model describing how an infection spreads within the colony when bee members engage in the trophallactic activity to distribute nectar. One possible application of our model is to explain the transmission of viruses within the colonies. Detection of viruses in hypopharyngeal glands of infected workers [16–18] implies trophallactic feeding as a transmission pathway from nurses to brood and among worker bees.

Many honeybee viruses have been documented to be involved in colony mortality. A metagenomic study revealed that the Israeli acute paralysis virus (IAPV) is strongly correlated with CCD-affected colonies in the US [68]. Chen et al. [16] reported a negative correlation between the level of IAPV infections and the size of infected colonies, which is also negatively associated with the overwintering mortality of the colonies. The presence of deformed wing virus (DWV) and acute bee paralysis virus (ABPV) is significantly related to winter losses of colonies in Switzerland [69] and Germany [70]. These viruses can persist in all developmental stages of honeybees at low levels without a noticeable impact on the individual and colony health. However, the viral infection accompanied by a high varroa infestation level can cause the viruses to become more virulent [56, 71–73]. *V. destructor* is necessary as a vector to overtly transmit DWV to pupae, causing pupal death and the emergence of inviable adult bees with a clinical symptom of deformed wings [73]. Our current model, however, did not include the mites. In fact, viral infection can impact on colony survival while the infection remains asymptomatic [16]. Our study demonstrated how subclinical infection could lead to colony losses in weak colonies (e.g., colonies with low laying rates). Nevertheless, explicitly adding mites and their role as a transmission vector into our future model will generate richer transmission dynamics as shown in previously published models [34–36, 74]. Subclinical infection, as simulated in our model, is also assumed to rule out the fecal-oral transmission as honeybees in healthy colonies normally defecate outside the hive.

2) The second area of concern is the assumption that immatures become infected under natural conditions. A few studies have shown that immatures can become infected under controlled/laboratory conditions. In addition, Urbieta-Margo et al. (2019) demonstrate natural infection, but at very low levels of infection using PCR methods. What remains to be shown is histological evidence of Nosema development within tissues of immatures collected from colonies.

Authors' response:

Our model is now more generalized toward viral infection, which can be detected in all developmental stages of honeybees, including immatures.

3) Furthermore, if it is accepted that immatures become infected, and that bees performing hygienic behavior remove infected immatures, then wouldn't the sequence of infection also be from immature to adult instead of only adult to

immature as per the model? It is common to see pupae that have been "chewed-down" midway through removal by hygienic bees. During this removal, if the immature was infected, then it would seem that adults performing removal may become infected by handling contaminated tissues. Perhaps the model should also be run without the inclusion of infected immatures and hygienic behavior acting on individuals in this stage?

Authors' response:
We modify Eq. 3 & 4 to include the probability of healthy nurses being infected from infected brood during performing the hygienic removal. Now the equations read:

$$\mathrm{N}' = \frac{\mathrm{B}}{n_{\mathrm{B}}} - \frac{\mathrm{N}}{n_{\mathrm{N}}} - p_{\mathrm{t1}} \cdot k_{\mathrm{RN}} \cdot \mathrm{iR}_1 \cdot \mathrm{N} - p_{\mathrm{t,rem}} \cdot k_{\mathrm{rem}} \cdot \mathrm{iB} \cdot \mathrm{N} \tag{3}$$

$$\mathrm{iN}' = \frac{\mathrm{B_i}}{n_{\mathrm{B}}} - \frac{\mathrm{iN}}{n_{\mathrm{N}}} + p_{\mathrm{t1}} \cdot k_{\mathrm{RN}} \cdot \mathrm{iR}_1 \cdot \mathrm{N} - k_{\mathrm{rem}} \cdot \mathrm{iN} \cdot \mathrm{N} + p_{\mathrm{t,rem}} \cdot k_{\mathrm{rem}} \cdot \mathrm{iB} \cdot \mathrm{N} - k_{\mathrm{d}} \cdot \mathrm{iN} \tag{4}$$

We explain the new terms in **Line 166–169**:
During infected brood removal by hygienic bees, workers performing removal may become infected by handling contaminated tissues with a probability $p_{t,rem}$ (Eq 3 and 4). The default value of $p_{t,rem}$ is 0.0 and we will explore this parameter later.

Then we explore the effect of $p_{t,rem}$ at **Line 229–237**:
In Fig 4, we explore two more parameters in our model, namely the probability of nurses being infected from infected brood during performing hygienic removal ($p_{t,rem}$) and the death rate of infected bees (k_d). The two-parameter bifurcation diagrams in Fig 4A and 4B reveal that as $p_{t,rem}$ increases, larger values of k_{rem} and I_0 are needed in maintaining the colony's health against an infection. In Fig 4C and 4D, k_d shows an opposite effect. As the death rate of infected bees increases, smaller values of k_{rem} and I_0 are required to maintain the colony in a healthy state. When the death rate becomes very high, the infection rapidly kills the infected bees, preventing further transmission to other nest-mates, and the colony is always in a healthy state.

And a new Figure 4 is added:

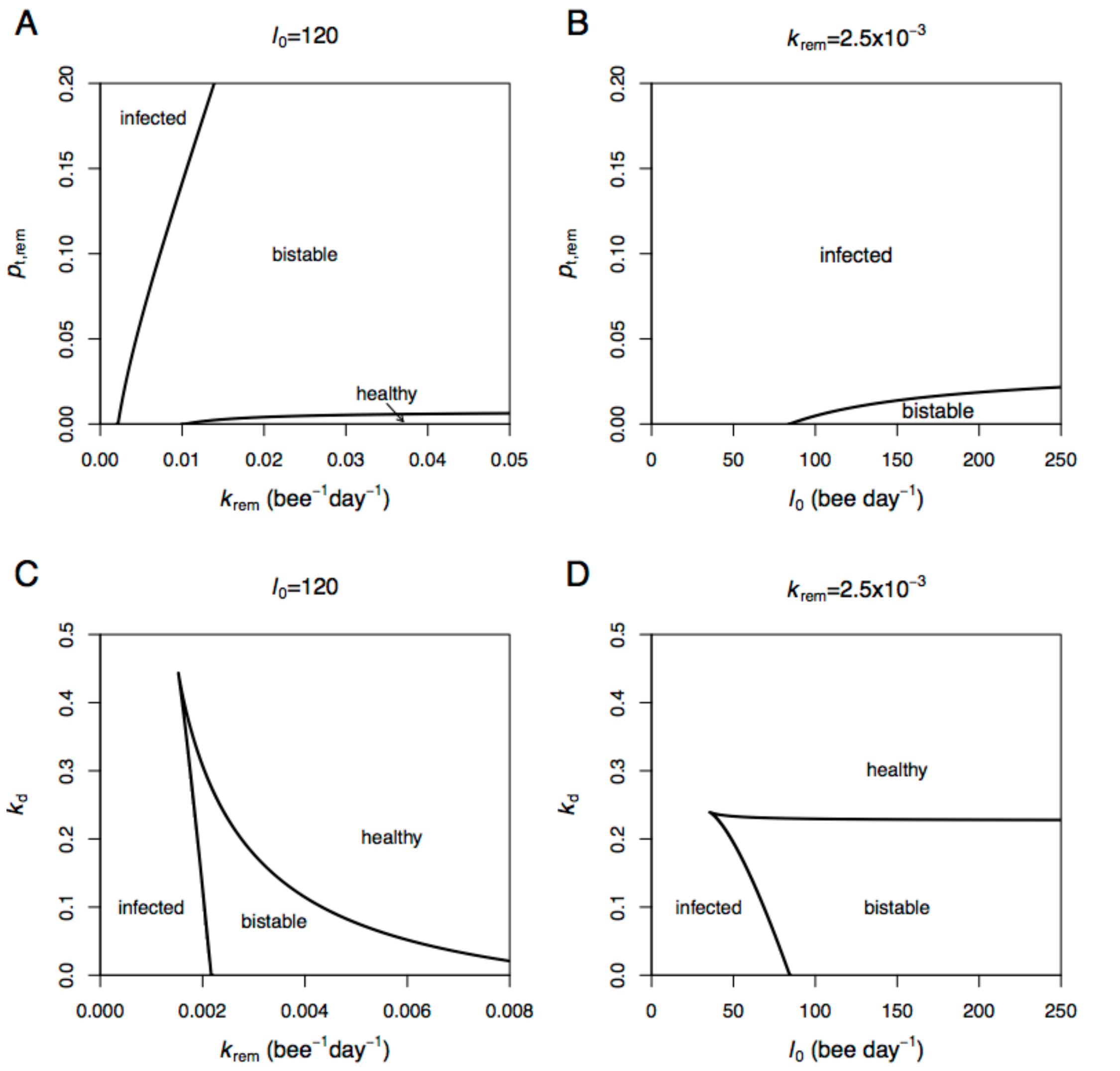

Fig 4. Role of the probability of nurses being infected from infected brood ($p_{t,rem}$) and the death rate of infected bees (k_d). The two-parameter bifurcation diagrams are plotted between A: k_{rem} and $p_{t,rem}$, B: I_0 and $p_{t,rem}$, C: k_{rem} and k_d, and D: I_0 and k_d. The diagrams are divided into regions corresponding to the state of the colony (healthy, bistable, and infected).

4) Minor Comments:
Overall, the manuscript is well-written. There are some language and usage issues; therefore, additional editing is recommended.

Authors' response:
We have used commercial software to grammatically correct the revised manuscript.

5) Line 5-6: Consider that some evidence shows stabilization or increase in honey bee populations worldwide (e.g., data from FAO).

Authors' response:
We revise our statement about 'bee declines' to also present the fact that the global number of managed colonies has risen.

Line 4–12:
However, large-scale losses of managed honeybee colonies have been reported in

some parts of North America and Europe in the past decades [3–7]. Additionally, in 2006, a special case of collapse in which adult worker bees rapidly disappear from colonies, leaving a large amount of brood to die, has been widely observed in the US. This phenomenon is referred to as Colony Collapse Disorder (CCD), and its exact causes remain unclear [3, 8]. Although the global number of managed colonies has risen by about 30% since 2000 [9], honeybee management has been challenged with increased pathogen and environmental pressure associating with increased beekeeping costs.

6) Line 58: APV is meant to be Acute bee paralysis virus (ABPV)?

Authors' response:
We now write 'ABPV' as suggested.

7) Line 250: It is not clear but does the model accommodate acceleration of behavioral development and premature death of non-infected bees responding to loss of infected bees?

Authors' response:
We did not implement the premature transition in our model. For the clarity, we state at **Line 346–348**:

Note that we did not implement the premature transition mechanism in the current version of our model as this behavior has already been a subject of investigation in many previous studies.

Reviewer #2 Comments

1) Review of A Model of *Nosema ceranae* Infection in Honeybee Colonies with Social Immunity, T. Laomettachit et al.

Overview: This paper develops and analyzes a new deterministic differential equation model, along with a related stochastic model, for *Nosema ceranae* disease dynamics within a honey-bee colony. The model divides the colony into four life stages (brood, nurses, nectar-receivers, and foragers), which effectively creates a socially segregated chain of disease transmission. The model also assumes that honeybees engage in a hygienic response by removing infected bees. Numerical experiments show that the social segregation and hygienic response, as well as colony size and seasonality, influence the risk of colony collapse.

General Comments: The authors present a relatively simple model of within-hive disease dynamics that elucidates the role of social immunity and hygienic behavior on honeybee colony survival or collapse. The assumptions and model parameters are rooted carefully in the biological literature, and the analysis is straightforward, thorough, and intuitively presented. Most of the results are expected, but some results are surprising (e.g., non-monotonic relationship between laying rate and colony collapse frequency). The manuscript is both succinct and thorough; it is effortless to understand and a pleasure to read.

I only have two major questions that need to be addressed before I recommend the manuscript be accepted. All other comments are minor, but addressing them would improve the manuscript.

Major Comments:
Extended model: Why are there no new forager infections (i.e., no transitions $F_0 \rightarrow iF_1$)? I suspect that the assumption of only one infected forager returning to the hive affects many of the presented results.

Authors' response:
We would like to express our appreciation for your insightful comments and suggestions.

The figure below compares our model (one infected forager is introduced $iF_1(0)=1$) with a model that allows returning foragers to be infected bees ($F_0 \rightarrow iF_1$) with a probability p_{inf}. The subfigures in the left panel are from the simulations of the original model and the subfigures in the right panel are from the simulations of the $F_0 \rightarrow iF_1$ transition model with p_{inf}=1E–5. It can be seen that the two models give agreeable results although there are some quantitative differences. In panel E and F, when the hygienic behavior is impaired (k_{rem}=0; red lines), the %survival reaches a minimum around 70% and 20% for the original model and the $F_0 \rightarrow iF_1$ transition model, respectively. When the homing ability is elevated (p_{surv}=0.5; blue lines), the original model yields the minimum %survival around 60% while the $F_0 \rightarrow iF_1$ transition model reaches the minimum %survival around 0%. (Note that the y-axis shows %survival rather than %collapse following your suggestion below.)

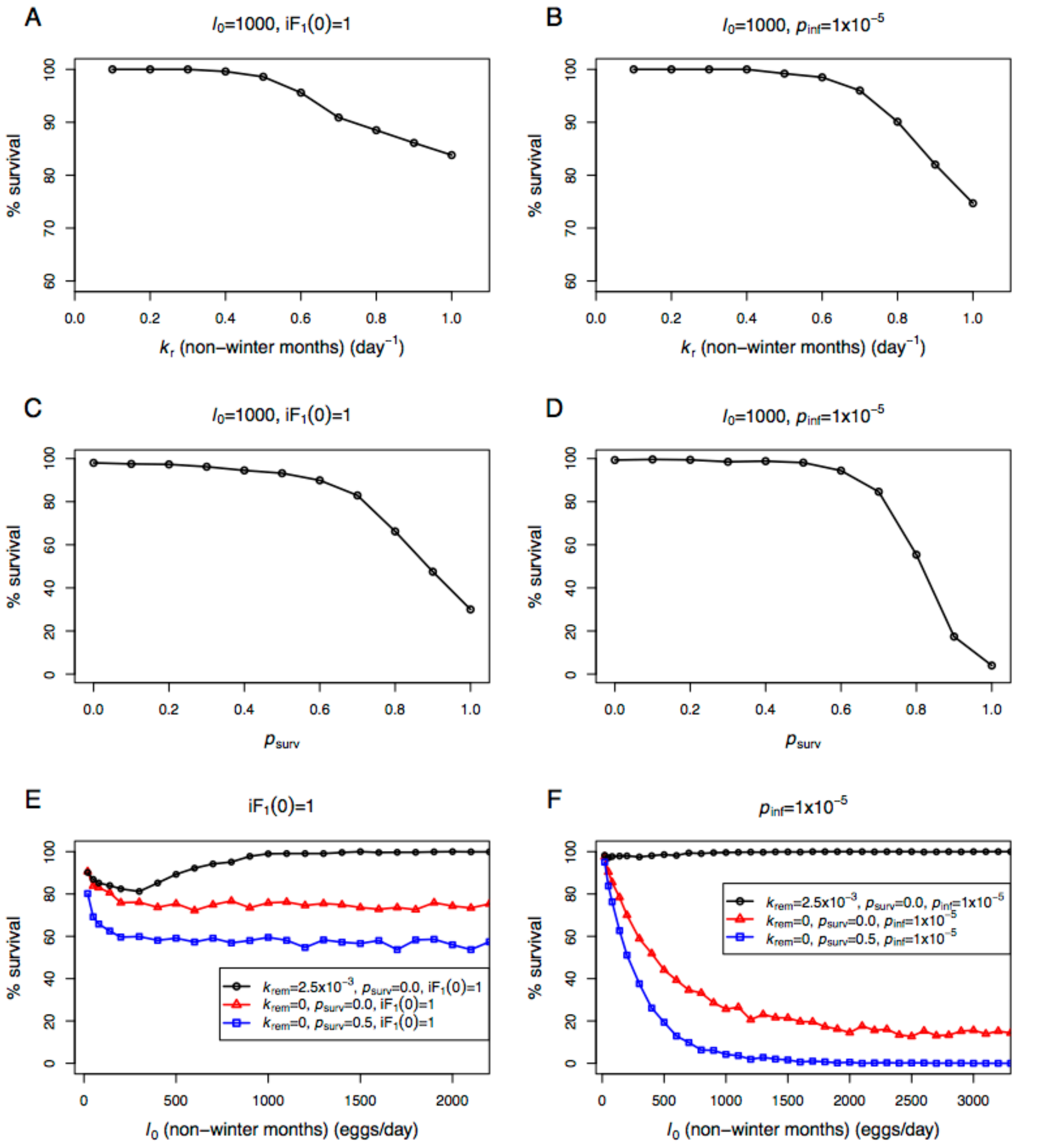

S3 Figure. Stochastic model with a constant probability of infection. The figure compares the model with an infection proposed in the main text (one infected forager is introduced at the onset of infection, iF$_1$(0)=1) (left panel) with the model that allows returning foragers to be infected bees ($F_0 \rightarrow iF_1$) with a probability p_{inf} (right panel). The two models are in qualitative agreement although quantitative differences can be seen.

The figure is included in the revised manuscript as Supplmentary Figure S3. The following paragraph is added into the main text.

Line **408–413**:

Infection in simulations in Fig 8 is introduced as one infected forager returning from nectar-collecting (iF$_1$) at the onset of spring. S3 Figure plots simulation results when an infection is introduced constantly over non-winter months. In the simulations, returning foragers have a probability p_{inf} of becoming infected. The plot shows that the results remain in qualitative agreement with those in Fig 8, although quantitative

differences can be seen.

2) pg 11, ln 360-371: The case $iR_1(0) = 1$ is only explored in an extreme circumstance (hygienic behavior and impaired homing both failed). What would happen if social segregation and impaired homing failed, but hygienic behavior remained (k_{rem} = 2.5e–3, p_{surv} = 0.5 and $iR_1(0) = 1$)? What would happen if social segregation failed, but impaired homing and hygienic behavior remained (k_{rem} = 2.5e–3, p_{surv} = 0 and $iR_1(0) = 1$)? I would trust the conclusions more if these cases were explored.

Authors' response:
We now include the suggested two cases in Figure 8C. (Note that the y-axis shows %survival rather than %collapse following your suggestion below.)

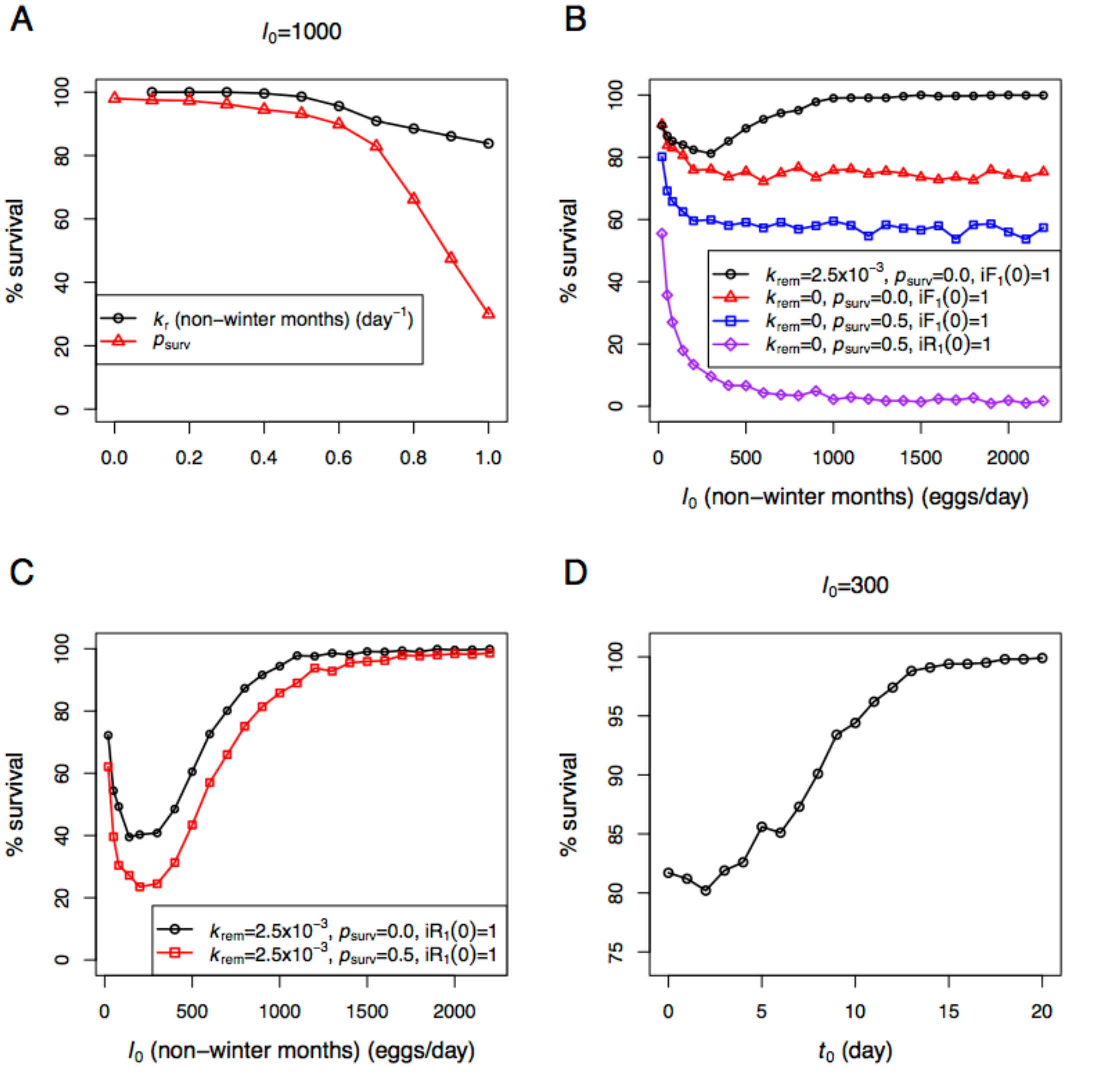

Fig 8. Percentages of survived colonies from stochastic simulations. Percentages of survived colonies are calculated from 1000 repeats of stochastic simulations. Model parameters are from Table 1, except those that are listed in each figure panel. Seasonal effects are implemented as described in the main text. A: k_r during non-winter months (black line) or p_{surv} (red line) is varied. B and C: I_0 during non-winter months is varied. D: The timing of an infection (t_0) is varied in relation to the onset of spring (i.e., $iF_1(t_0)=1$).

The following paragraph is added to the main text.

Line **391–400**:

Fig 8C explores two more cases in which social segregation is compromised (one infected nectar-receiver (iR_1) is introduced in early spring and p_{surv}=0.0 or 0.5), but with intact hygienic behavior ($k_{rem}=2.5 \times 10^{-3}$). The plot shows that without the social segregation mechanism, colonies suffer severe losses as the laying rate initially increases. However, colonies with higher laying rates can effectively mitigate the losses. Therefore, it becomes evident from our simulations that organizational immunity in terms of social segregation combined with impaired homing ability of infected foragers forms the first line of defense to reduce the uptake of pathogens into the colony. If this first line of defense fails, the hygienic behavior ($k_{rem}>0$) provides another mechanism in the prevention of disease spread within the colony.

3) Minor Comments:
pg 2, ln 27-29: What is the biological mechanism responsible for reduced lifespan, reduced colony size, reduced honey production, and increased mortality? Knowing the mechanism will help the reader assess the plausibility of model assumptions.

Authors' response:
As suggested by other reviewers, the claims about our model being specific to *Nosema ceranae* is replaced with a more general model and the sentences the reviewer mentioned were removed.

4) pg 4, ln 115-117: What is the biological mechanism for infection detection and removal? Knowing this will also help the reader assess the plausibility of model assumptions.

Authors' response:
We add a possible biological mechanism at **Line 120–122**:

The recognition of infected individuals by bees performing hygienic behavior is triggered by olfactory cues, e.g., by detecting cuticular hydrocarbon changes in sick bees [32, 52].

5) Core model: Is there biological evidence for maturation while infected without any significant disease mortality? Disease mortality is mentioned much later on pg 8, ln 250-253, and I think this should be moved into the core model section.

Authors' response:
As suggested by other reviewers, we remove the claims about our model being a specific case for *Nosema ceranae*. Instead, we now present our model as a more general model to a communicable disease transmitted through trophallaxis with a possible application to explain viral transmission within the colonies.

We modify Eq 2 and 4 to include the death of infected bees and discuss infection mortality in the core model section.

$$\mathrm{iB}' = -\frac{\mathrm{iB}}{n_{\mathrm{B}}} + p_{\mathrm{t0}} \cdot k_{\mathrm{NB}} \cdot \mathrm{iN} \cdot \mathrm{B} - k_{\mathrm{rem}} \cdot \mathrm{iB} \cdot \mathrm{N} - k_{\mathrm{d}} \cdot \mathrm{iB} \qquad (2)$$

$$\mathrm{iN}' = \frac{\mathrm{B_i}}{n_\mathrm{B}} - \frac{\mathrm{iN}}{n_\mathrm{N}} + p_{\mathrm{t1}} \cdot k_{\mathrm{RN}} \cdot \mathrm{iR_1} \cdot \mathrm{N} - k_{\mathrm{rem}} \cdot \mathrm{iN} \cdot \mathrm{N} + p_{\mathrm{t,rem}} \cdot k_{\mathrm{rem}} \cdot \mathrm{iB} \cdot \mathrm{N} - k_\mathrm{d} \cdot \mathrm{iN} \quad (4)$$

Line 170–177:
Infection of individual honeybees results in variable outcomes depending on the infection loads and other stress factors. For example, most viruses infect honeybees without clinical symptoms [56], but the infection can become acute following other stresses, leading to honeybee mortality. We set the default value of k_d = 0.0 per day to represent subclinical infection. Later, we will vary the parameter to explore for lethal infection effects on the colony status. (We assume both infected brood and workers have the same death rate, k_d, for simplicity (Eq 2 and 4)).

We then explore the effect of k_d at **Line 229–237**:

In Fig 4, we explore two more parameters in our model, namely the probability of nurses being infected from infected brood during performing hygienic removal ($p_{\mathrm{t,rem}}$) and the death rate of infected bees (k_d). The two-parameter bifurcation diagrams in Fig 4A and 4B reveal that as $p_{\mathrm{t,rem}}$ increases, larger values of k_{rem} and I_0 are needed in maintaining the colony's health against an infection. In Fig 4C and 4D, k_d shows an opposite effect. As the death rate of infected bees increases, smaller values of k_{rem} and I_0 are required to maintain the colony in a healthy state. When the death rate becomes very high, the infection rapidly kills the infected bees, preventing further transmission to other nest-mates, and the colony is always in a healthy state.

And a new Figure 4 is added:

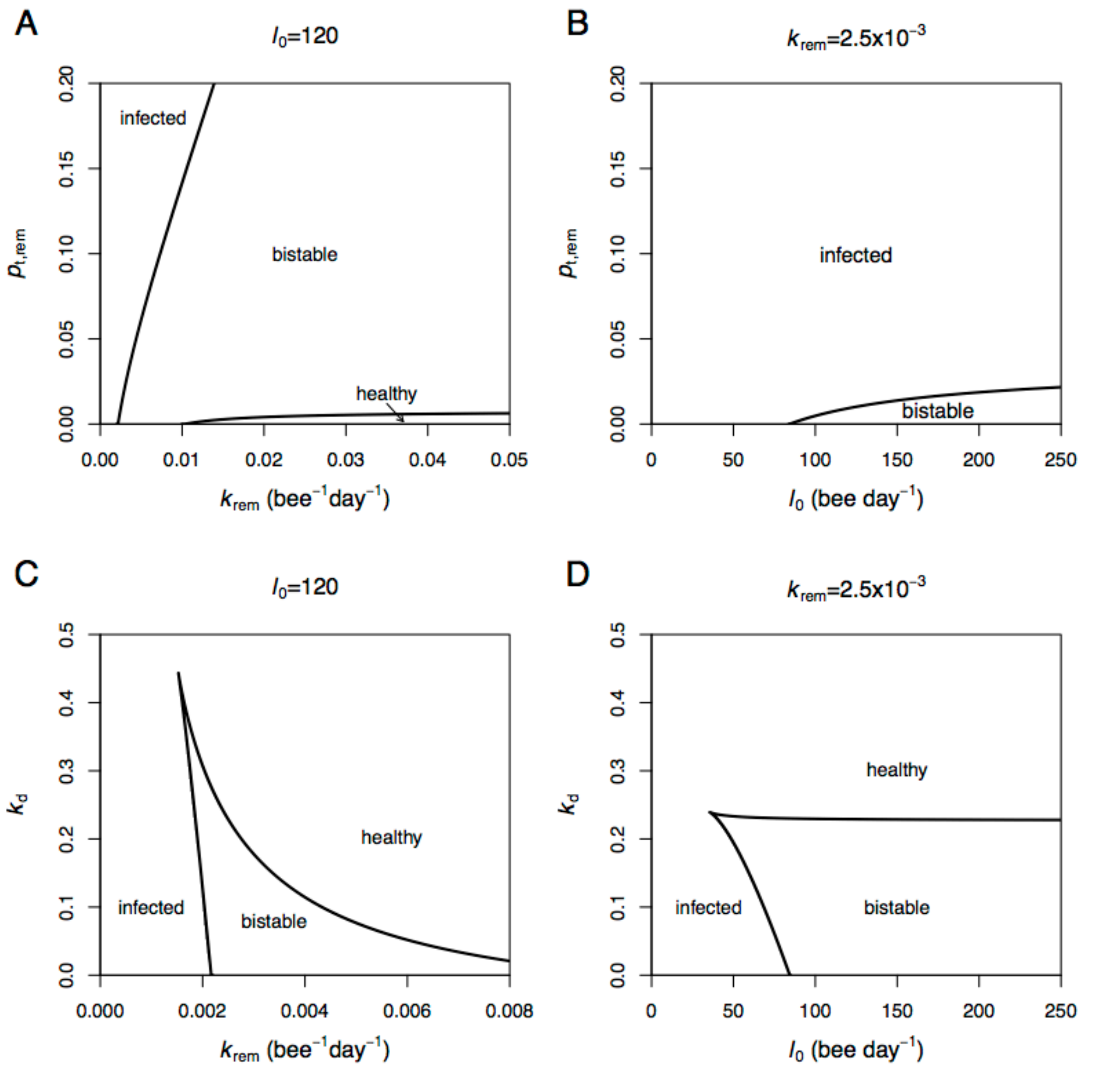

Fig 4. Role of the probability of nurses being infected from infected brood ($p_{t,rem}$) and the death rate of infected bees (k_d). The two-parameter bifurcation diagrams are plotted between A: k_{rem} and $p_{t,rem}$, B: I_0 and $p_{t,rem}$, C: k_{rem} and k_d, and D: I_0 and k_d. The diagrams are divided into regions corresponding to the state of the colony (healthy, bistable, and infected).

6) pg 5, ln 132-133: Please cite the references from Table 1 when claiming n_B = 20 and n_N = 10.

Authors' response:
Edited as suggested.

7) Table 1: It is assumed that transmission probabilities given contact are identical for the brood, nurses and nectar-receivers. Is there biological evidence for this assumption, or is it made for simplicity?

Authors' response:
The transmission probabilities are assumed to be identical just for the sake of simplicity.

8) pg 5, ln 145: Is there no chance a nurse will get infected in the removal process? I imagine that killing an infected bee might risk transmission.

**Authors' response:**<br>To include the suggested transmission, we modify Eq. 3 & 4 to include the probability of healthy nurses being infected from infected brood during performing hygienic removal. Now the equations read:<br>$$\mathrm{N}' = \frac{\mathrm{B}}{n_\mathrm{B}} - \frac{\mathrm{N}}{n_\mathrm{N}} - p_{\mathrm{t1}} \cdot k_{\mathrm{RN}} \cdot \mathrm{iR}_1 \cdot \mathrm{N} - p_{\mathrm{t,rem}} \cdot k_{\mathrm{rem}} \cdot \mathrm{iB} \cdot \mathrm{N} \quad (3)$$<br>$$\mathrm{iN}' = \frac{\mathrm{B_i}}{n_\mathrm{B}} - \frac{\mathrm{iN}}{n_\mathrm{N}} + p_{\mathrm{t1}} \cdot k_{\mathrm{RN}} \cdot \mathrm{iR}_1 \cdot \mathrm{N} - k_{\mathrm{rem}} \cdot \mathrm{iN} \cdot \mathrm{N} + p_{\mathrm{t,rem}} \cdot k_{\mathrm{rem}} \cdot \mathrm{iB} \cdot \mathrm{N} - k_\mathrm{d} \cdot \mathrm{iN} \quad (4)$$<br>We explain the new terms in **Line 166–169**:<br>During infected brood removal by hygienic bees, workers performing removal may become infected by handling contaminated tissues with a probability $p_{t,rem}$ (Eq 3 and 4). The default value of $p_{t,rem}$ is 0.0 and we will explore this parameter later.<br><br>Then we explore the effect of $p_{t,rem}$ along with the effect of k_d at **Line 229–237** and in the newly added Fig 4 (please see our response #5 above).
9) The model also assumes that infected nurses would not attempt to remove infected brood or workers. Is this know, and if so, what is the biological mechanism preventing infected nurses from enacting hygienic behavior?
**Authors' response:**<br>The assumption is made just for simplicity of the model.
10) pg 5, ln 161-162: In the core model, $p_{t1} k_{RN}$ iR_1 = 5e–4. Why is this selection made?
**Authors' response:**<br>The value of 5E–4 was selected so that the core model behaves in agreement with the full model. Specifically, the selected value allows both core and full models to make a critical transition via a saddle-node bifurcation from the healthy to infected state when l_0 decreases below around 84.
11) Figure 2: How are these figures generated (analytical or numerical)?
**Authors' response:**<br>The equations are solved numerically. We add the following at **Line 178–179**:<br>With model parameters listed in Table 1, the equations are solved numerically with XPPAUT (http://www.math.pitt.edu/~bard/xpp/xpp.html).
12) Figure 3: Laying rate is used as a proxy for colony size, and while a larger laying rate will under most circumstances lead to larger colony size, I find it a bit misleading to use laying rate and colony size (or brood size or nurse numbers) nearly interchangeably.
**Authors' response:**<br>In the revised manuscript, we replace 'colony size' with 'egg-laying rate' in places

where it is appropriate.

13) Figure 3: A bifurcation diagram of $B(0)$ versus $N(0)$ may be informative in the bistable region. I suspect the ratio of brood to nurses is important.

Authors' response:
We show in Fig 3 C & D that not only the ratio of brood to nurses is important, but also the total numbers of both classes.

14) Figure 3: In subfigure B, consider removing the cross and asterisk markers in favor of an arrow or letters. Asterisks imply a bifurcation point in MatCont, and I found it confusing.

Authors' response:
We now use letters as markers in Fig 3B.

15) pg 8, ln 227-228: Please cite the references from Table 1 when claiming $n_R = 11$ and $n_F = 14$.

Authors' response:
Edited as suggested.

16) pg 9, ln 287-289: Would model results drastically change if the laying rate were explicitly dependent on foraging?

Authors' response:
Adding the suggested feature will provide richer dynamics to the system. We hope to add this feature into our future version of the model.

17) pg 9-10, ln 295-298: Is there biological evidence that larger colonies are less likely to collapse due to disease?

Authors' response:
We provide evidence by referring to the study by Chen et al. 2014 (PLOS Pathog. 2014;10(7):e1004261). We discuss about the evidence at **Line 470–471**:
Our finding is in concert with Chen et al. [16], who observed that large colonies suffer lower IAPV infection levels than small colonies.

And at **Line 481–483**:
This finding is in line with a study observing that all large colonies infected with IAPV survived through the winter months, while almost all small colonies infected with IAPV died before spring [16].

18) pg 10, ln 316: What is the logic behind the 0.8 infection threshold? Is there biological evidence that a hive will collapse once 80% of the colony is infected, or was this an arbitrary threshold?

Authors' response:
Our model exhibits collapse when the colony makes a critical transition from the healthy to infected state via a saddle-node bifurcation (i.e., almost all bees suddenly become infected). Therefore, the infection threshold is arbitrary, but it needs to be large enough to avoid transient fluctuation of the number of infected individuals

around the critical point. Any infection thresholds that are large enough should give the same results when counting the number of collapsed colonies.

19) pg 11, ln 337-338: Please explain the intuition behind the impaired hygienic response for moderate laying rates. I did not understand precisely which model assumption(s) led to this result.

Authors' response:
The hygienic response is more effective in large colonies than small colonies. This is due to a higher frequency of interactions between nurses and infected honeybees in large colonies. However, simulations of our full model reveal that colonies with moderate laying rates (e.g., l_0=200) have the lowest % survival. This is due to the interplay between pathogens' exploitation of frequent transmission interactions among individuals and the effectiveness of the hygienic response by nurses in large colonies. We revise our explanation in **Line 354–363** to clarify this point.

Line 354–363:
In stochastic simulations (Fig 8B, black line) the percentage of colony survival is found to decrease as the rate of egg-laying initially increases (l_0 from 50 to 200), but later increase as the laying rate becomes higher (l_0>200). As the rate of egg-laying initially increases, the population size also increases, which associates with a higher probability of disease transmission due to a higher frequency of interactions between individuals. Therefore, from l_0 equal to 50 to 200, the survival percentage gradually drops. In previous sections, we have shown that larger colonies are more effective in performing the hygienic response. Therefore, as the laying rate becomes very high (l_0>200), the rate at which infected bees are removed gradually exceeds the rate of transmission; thus, the survival is more observed.

20) Figure 7: These figures would be easier to digest if the *y*-axis were % survival rather than % collapse. All previous figures had survival on the *y*-axis.

Authors' response:
The figure (Fig 8 in the revised manuscript) now represents % survival on the y-axis.

21) Starting in the Results section, the manuscript contains several grammatical errors and typos. None impair understanding except on pg 12, ln 405.

Authors' response:
We have used commercial software to grammatically correct the revised manuscript. We edit line 405 mentioned by the reviewer to:

Line 459–461:
We demonstrated with stochastic simulations that the social segregation, combined with the impaired homing ability of infected foragers, limits the risk of pathogen uptake into the colony below a certain level.

22) pg 13, ln 465-475: For the sake of flow, consider moving this paragraph before the "In summary..." paragraph.

Authors' response:
The paragraphs are rearranged as suggested.

Reviewer #3 Comments

1) Summary of Comments

Main Comments

This is one of many technically good mathematical models attempting to understand the dynamics and infection processes underpinning honey bee colony loss. Whilst the authors' capturing of interaction structure and social hygienic behaviours is of interest, worthy of pursuit, and will contribute to the field, the current paper sits an awkward place of alleging to be pathogen specific without doing an adequate job of capturing that pathogen's biology (see some points below). However, the presentation of the manuscript lacks generality or reference to which other pathogens it might represent, and where it sits comparatively with other demographic or epidemiological models. I have given some discussion below as to my objections to some of the core biology underpinning the model, which will need revision.

I do believe this work will be useful, but I recommend the authors commit to either: 1) a *Nosema ceranae* specific model with much greater exploration of the true biology of the pathogen, including its density-dependent airborne transmission and facultative precocial foraging by bees upon infection; or 2) a much more generic model where they explore parameter space more generally, remove their claims to this being a specific study of the Nosema system, and more generally overview which pathogens exhibit biology close to what they are modelling.

Authors' response:

We would like to express our appreciation for your insightful comments.

We remove the claims about our model being a specific case for *Nosema ceranae*. As suggested, we now present our work as a more general model to a communicable disease transmitted through trophallaxis with a possible application to explain viral transmission within the colonies. We also change the title of our manuscript to 'A Model of Infection in Honeybee Colonies with Social Immunity'.

To justify why oral-oral transmission is the preferred route, we add a new paragraph discussing the role of trophallaxis in viral transmission.

Line 20–31:

Honeybees living in large colonies are prone to the rapid spreading of pathogens among individuals due to high population density and high contact rates. Trophallaxis (mouth-to-mouth food sharing) is considered a routine behavior that facilitates pathogen transmission [12, 13]. For example, trophallaxis may be the predominant mechanism of horizontal viral transmission. Nurse bees infected with viruses can transmit them to the larvae via trophallaxis since they rely heavily on nurses' tending and feeding. The transmission route is supported by the detection of viruses, such as deformed wing virus (DWV) [14], sacbrood virus (SBV) [15], and Israeli acute paralysis virus (IAPV) [16], in larval food. In addition, the detection of viruses in the hypopharyngeal gland of infected worker bees was demonstrated for acute bee paralysis virus (ABPV) [17], sacbrood virus [18], and Israeli acute paralysis virus (IAPV) [16], implying a possible foodborne transmission route driven by trophallaxis.

In the Discussion section, we point out a possible application of our model to explain viral transmission.

Line 418–444:

We developed a generic model describing how an infection spreads within the colony when bee members engage in the trophallactic activity to distribute nectar. One possible application of our model is to explain the transmission of viruses within the colonies. Detection of viruses in hypopharyngeal glands of infected workers [16–18] implies trophallactic feeding as a transmission pathway from nurses to brood and among worker bees.

Many honeybee viruses have been documented to be involved in colony mortality. A metagenomic study revealed that the Israeli acute paralysis virus (IAPV) is strongly correlated with CCD-affected colonies in the US [68]. Chen et al. [16] reported a negative correlation between the level of IAPV infections and the size of infected colonies, which is also negatively associated with the overwintering mortality of the colonies. The presence of deformed wing virus (DWV) and acute bee paralysis virus (ABPV) is significantly related to winter losses of colonies in Switzerland [69] and Germany [70]. These viruses can persist in all developmental stages of honeybees at low levels without a noticeable impact on the individual and colony health. However, the viral infection accompanied by a high varroa infestation level can cause the viruses to become more virulent [56, 71–73]. *V. destructor* is necessary as a vector to overtly transmit DWV to pupae, causing pupal death and the emergence of inviable adult bees with a clinical symptom of deformed wings [73]. Our current model, however, did not include the mites. In fact, viral infection can impact on colony survival while the infection remains asymptomatic [16]. Our study demonstrated how subclinical infection could lead to colony losses in weak colonies (e.g., colonies with low laying rates). Nevertheless, explicitly adding mites and their role as a transmission vector into our future model will generate richer transmission dynamics as shown in previously published models [34–36, 74]. Subclinical infection, as simulated in our model, is also assumed to rule out the fecal-oral transmission as honeybees in healthy colonies normally defecate outside the hive.

2) I also believe they should better explicitly reference what their model does similarly and differently to other models in this system, some of which they reference well, others poorly, and some are missed.

Authors' response:

We now point out the differences of our model to other models in the Introduction section.

Line 83–98:

The present model has several key features that are different from other previous models. In previous models, the role of social immunity, particularly the social segregation between low-risk and high-risk bees and the hygienic behavior toward sick bees, has not been the main focus before (except a couple of models, see [24] [45]). We incorporate the two features into our model. First, our model implements social segregation. High-risk individuals such as foragers are limited to contact only nectar-receivers, but not other vulnerable individuals (nurses and brood) inside the nest. Second, our model includes the hygienic behavior, by which healthy nurses

actively remove infected workers and brood from the colony. The two features constitute the social immunity in our model and play a crucial role in preventing horizontal transmission of pathogens from the infected ones to other nest-mates.

Additionally, previous models were deterministic-based (except [24] and [44]), neglecting the stochastic nature of infection transmission. Our study emphasizes the role of stochasticity that contributes to colony status. The conditions that do not cause colony death in a deterministic setting can cause colony mortality in our stochastic simulations.

3) Introduction
The introduction is long for the paper and will likely require some changes following my recommendations below.

In particular, Lines 1-21: This section reads as extremely generic and, in places, it is incorrect. For example, 'CCD' has been well hypothesised as a symptom of elevated forager mortality following honey bee modelling work by Khoury et al 2011, work which the authors do cite later in the introduction but without this clarity. Much of the reference to CCD, which is no longer considered a useful term by honey bee biologists, seems to be done as a generic replacement for colony death (precisely why we now discourage its use). The authors state that worldwide, colonies are declining (Line 5). This is simply incorrect (see UN FAO data showing worldwide increase in honey bee colonies despite declines in parts of NW Europe and North America).

Authors' response:
We now avoid using "CCD" as a blanket for all colony losses in our revised manuscript. We also revise our statement about 'bee declines' to also present the fact that the global number of managed colonies has risen.

Line 4–12:
However, large-scale losses of managed honeybee colonies have been reported in some parts of North America and Europe in the past decades [3–7]. Additionally, in 2006, a special case of collapse in which adult worker bees rapidly disappear from colonies, leaving a large amount of brood to die, has been widely observed in the US. This phenomenon is referred to as Colony Collapse Disorder (CCD), and its exact causes remain unclear [3, 8]. Although the global number of managed colonies has risen by about 30% since 2000 [9], honeybee management has been challenged with increased pathogen and environmental pressure associating with increased beekeeping costs.

4) I also believe the authors miss some critical studies honey bee mathematical modelling which their work should be framed against, for example Booton et al 2017 https://www.sciencedirect.com/science/article/abs/pii/S0022519317301212 and Bartlett et al and Bartlett et al 2019
https://besjournals.onlinelibrary.wiley.com/doi/full/10.1111/1365-2664.13461 both of which include aspects of this work which are, to my view, missed.

Authors' response:
We now add both references in the Introduction section.

Line 61-65:
Booton et al. [40] extended Khoury's model frameworks to include density-dependent mortality of worker bees. The model predicted that a small change in the rate of regulatory processes such as forager recruitment, social inhibition, and egg-laying could cause sudden colony losses due to a critical transition via a saddle-node bifurcation.

Line 72-75:
Modeling inter-colony pathogen transmission has also been presented. Bartlett et al. [44] examined how management practices (e.g., colony numbers and colony arrangement configurations) at the apiary scale could impact pathogen prevalence.

5) For theory to remain cohesive and integrated within the field of honey bee biology, more explicit reference must be made to similarities and differences between model studies.

I would ask the authors to explicitly state in the introduction which parts of other models they capture, which they do not, and what they introduce which is new in the context of honey bee mathematical modelling.

Authors' response:
We now point out the new features included in our model that are different from other models in the Introduction section (**Line 83–98)** (Please see our response #2). We also discuss the features that our current model did not capture in the Conclusion section.

Line 497–505:
Our model can be extended in several ways. Social processes and regulations such as social inhibition (foragers slow down hive-to-forager development) and cooperative brood rearing by worker bees were ignored in the present model. These processes are of important factors determining the colony's fate, as proposed in [37–39]. These processes can be incorporated into the future version of our model to explore their interplay with the social immunity mechanisms. Also, behavioral changes of individual bees upon infection could be examined. For example, IAPV-infected bees were observed to engage less in trophallaxis with other nest-mates, presumably, to reduce pathogen transmission [76].

6) Methods, Results, Discussion

The results and discussion are well presented and seem to my reading correct in their derivations. However, I do not believe the core biological assumptions made can be justified.

For example, the authors themselves state that there is no evidence of the removal of Nosema infected brood, but include it regardless. I do not object to the modelling in principle, but the study should not be presented as a study of a specific disease system if it does not reflect the known biology.

On this topic, it has been experimentally shown that Nosema infections can occur without trophallaxis (a frequency-dependant infection dynamic) simply via close, non-contact proximity (and that in this regard, it is a density-dependant infection

dynamic). Roberts & Hughes 2015 (https://www.cambridge.org/core/journals/parasitology/article/horizontal-transmission-of-a-parasite-is-influenced-by-infected-host-phenotype-and-density/B05BBB47CE18A0A7F8323489F2C322FC) showed this specifically for Nosema ceranae in honeybees. Given this, I do not believe the model suitable for the specific studying of Nosema infections in honey bee colonies. If the authors wish to remain specifically studying Nosema, I believe the model must be revised. If they wish to present this as a generic model including both infected culling and infection purely via direct interaction then they must justify this biologically and remove explicit claims of modelling Nosema.
**Authors' response:**<br>We remove the claims about our model being a specific case for *Nosema ceranae* and now present our work as a more general model to a communicable disease transmitted through trophallaxis (please see our response #1).
7) This extends to their citing of Goblirsch et al for the additional mortality at the foraging stage of Nosema infection. Goblirsch et al explicitly state that infection with Nosema induces preferential abandonment of in-colony duties and a precocial foraging, which would have serious implications for their models behaviour and the conclusions to be drawn from it. I realise the authors mention this extension in line 473-475, but I believe the importance of this point to justifies its inclusion in the original model.<br><br>I do however believe the model framework in how it addresses hygienic behaviour & social culling, forager/ receiver / onward transmission, and the implications thereof to be strong and worth publishing with modification, although I do also believe that a flexible task allocation and infection-dependant precocial ageing into the forager class should be included in this (see above reference and many others).
**Authors' response:**<br>As we now present our work as a more general model, we do not implement the premature transition mechanism in our current model. We hope to explore this feature and its interplay with the social immunity in the future version of our model.
8) I have not given detailed comments beyond this, as I consider these biological problems with the model's mandate to require enough revision that additional commentary on more detailed aspects of the work to not remain relevant. However, I would be happy grateful to review this manuscript again in more detail if the authors agree with some of these recommendations.
**Authors' response:**<br>We hope that our revision addresses concerns and recommendations raised by the reviewer.
9) Conclusion<br>Similarly, I expect part of the conclusion to change if the biological rationale is improved. I also mirror my comments regarding the introduction that the conclusion is very generic in its discussion around compound stressors and alleged 'CCD', for example Lines 433-445 have little relevance to the work here as they are currently written. If the authors wish to discuss a variety of pathogens of honeybees, it should

be done with regards to their known biology, and whether the interaction structure and social hygienic behaviours they present would behave similarly or differently.
**Authors' response:**<br>We remove the sentences that are too generic and irrelevant to the current model. We now only discuss the queen's age and its possible effects on the colony status.<br><br>**Line 484-488**:<br>Additionally, as the egg-laying capacity declines gradually as the queens age, we predict that colonies headed by old queens may have a lower chance of overwintering survival. Consistently, the queen age was identified among multiple other factors, including mite infestation, DWV infection, and ABPV infection, to contribute to winter colony losses in Germany [70].
10) It is critical the authors make it clear what parasites they believe these findings to apply to, and which they do not. Note I do not believe this model currently approximates Nosema biology well enough to be stated as a study of Nosema ceranae infection dynamics.
**Authors' response:**<br>We now present our work as a more general model with a possible application to viral infection (please see our response #1).
