# Supplementary material for: A model of infection in honeybee colonies with social immunity
Source: PLoS One. 2021 Feb 22;16(2):e0247294. doi: 10.1371/journal.pone.0247294 (PMC7899363; doi:10.1371/journal.pone.0247294)
Supplement: Peer review history

# Peer review: Decision Letter 0

**DOI:** 10.1371/journal.pone.0247294.r001

**Authors:** Adam G Dolezal

28 Sep 2020

PONE-D-20-25709

A Model of Nosema ceranae Infection in Honeybee Colonies with Social Immunity

PLOS ONE

Dear Dr. Laomettachit,

Thank you for submitting your manuscript to PLOS ONE. After careful consideration, we feel that it has merit but does not fully meet PLOS ONE’s publication criteria as it currently stands. Therefore, we invite you to submit a revised version of the manuscript that addresses the points raised during the review process.

Please submit your revised manuscript by Nov 12 2020 11:59PM. If you will need more time than this to complete your revisions, please reply to this message or contact the journal office at plosone@plos.org. When you're ready to submit your revision, log on to https://www.editorialmanager.com/pone/ and select the 'Submissions Needing Revision' folder to locate your manuscript file.

Please include the following items when submitting your revised manuscript:

- A rebuttal letter that responds to each point raised by the academic editor and reviewer(s). You should upload this letter as a separate file labeled 'Response to Reviewers'.
- A marked-up copy of your manuscript that highlights changes made to the original version. You should upload this as a separate file labeled 'Revised Manuscript with Track Changes'.
- An unmarked version of your revised paper without tracked changes. You should upload this as a separate file labeled 'Manuscript'.

If you would like to make changes to your financial disclosure, please include your updated statement in your cover letter. Guidelines for resubmitting your figure files are available below the reviewer comments at the end of this letter.

If applicable, we recommend that you deposit your laboratory protocols in protocols.io to enhance the reproducibility of your results. Protocols.io assigns your protocol its own identifier (DOI) so that it can be cited independently in the future. For instructions see: http://journals.plos.org/plosone/s/submission-guidelines#loc-laboratory-protocols

We look forward to receiving your revised manuscript.

Kind regards,

Adam G Dolezal

Academic Editor

PLOS ONE

Journal Requirements:

When submitting your revision, we need you to address these additional requirements.

1. Please ensure that your manuscript meets PLOS ONE's style requirements, including those for file naming. The PLOS ONE style templates can be found at

https://journals.plos.org/plosone/s/file?id=wjVg/PLOSOne_formatting_sample_main_body.pdf and

https://journals.plos.org/plosone/s/file?id=ba62/PLOSOne_formatting_sample_title_authors_affiliations.pdf

2.Thank you for stating the following financial disclosure:

 [T.L. and O.D. acknowledge the Thailand Research Fund (TRF) and King Mongkut's University of Technology Thonburi (KMUTT) under Contract Number MRG5980039 for providing the financial supports. The funders had no role in study design, data analysis, decision to publish, or preparation of the manuscript.].

We note that one or more of the authors is affiliated with the funding organization, indicating the funder may have had some role in the design, data collection, analysis or preparation of your manuscript for publication; in other words, the funder played an indirect role through the participation of the co-authors. If the funding organization did not play a role in the study design, data collection and analysis, decision to publish, or preparation of the manuscript and only provided financial support in the form of authors' salaries and/or research materials, please do the following:

1. Review your statements relating to the author contributions, and ensure you have specifically and accurately indicated the role(s) that these authors had in your study. These amendments should be made in the online form.

1. Confirm in your cover letter that you agree with the following statement, and we will change the online submission form on your behalf:

“The funder provided support in the form of salaries for authors [insert relevant initials], but did not have any additional role in the study design, data collection and analysis, decision to publish, or preparation of the manuscript. The specific roles of these authors are articulated in the ‘author contributions’ section.”

Additional Editor Comments (if provided):

As you can see from the reviews below, each reviewer thought this manuscript has the potential to provide a valuable contribution to the field and has many positive aspects. However, each of the reviewers also brought up important points that will need addressing or revision.

In particular, there are several issues with assumptions based on the known biology of honey bees and Nosema infection, many of which are pointed out by the reviewers. I recommend the authors carefully address these points in the revision. I feel that the reviewers have provided extremely helpful advice, with references, to assist the authors in these revisions.

I will also highlight Reviewer #3's note about the use of "CCD" as a blanket for all colony deaths/bee losses. This term has a very specific definition that quickly became diluted by misuse and is thus only used in current honey bee research literature under very specific conditions. They also highlight the care needed in noting 'bee declines' as they are correct in their point that colony numbers are not declining in the last decade (some areas, like the USA, have seen declines in numbers since the mid-20th century, but these changes are due to complex factors). Instead, I recommend the authors look into data on colony losses as a component of beekeeping costs; i.e., beekeepers require greater monetary input to keep colony numbers at the needed level.

[Note: HTML markup is below. Please do not edit.]

Reviewers' comments:

Reviewer's Responses to Questions

**Comments to the Author**

1. Is the manuscript technically sound, and do the data support the conclusions?

The manuscript must describe a technically sound piece of scientific research with data that supports the conclusions. Experiments must have been conducted rigorously, with appropriate controls, replication, and sample sizes. The conclusions must be drawn appropriately based on the data presented. 

Reviewer #1: Yes

Reviewer #2: Yes

Reviewer #3: No

**********

2. Has the statistical analysis been performed appropriately and rigorously? 

Reviewer #1: N/A

Reviewer #2: N/A

Reviewer #3: N/A

**********

3. Have the authors made all data underlying the findings in their manuscript fully available?

The PLOS Data policy requires authors to make all data underlying the findings described in their manuscript fully available without restriction, with rare exception (please refer to the Data Availability Statement in the manuscript PDF file). The data should be provided as part of the manuscript or its supporting information, or deposited to a public repository. For example, in addition to summary statistics, the data points behind means, medians and variance measures should be available. If there are restrictions on publicly sharing data—e.g. participant privacy or use of data from a third party—those must be specified.

Reviewer #1: Yes

Reviewer #2: Yes

Reviewer #3: Yes

**********

4. Is the manuscript presented in an intelligible fashion and written in standard English?

PLOS ONE does not copyedit accepted manuscripts, so the language in submitted articles must be clear, correct, and unambiguous. Any typographical or grammatical errors should be corrected at revision, so please note any specific errors here.

Reviewer #1: Yes

Reviewer #2: Yes

Reviewer #3: Yes

**********

5. Review Comments to the Author

Please use the space provided to explain your answers to the questions above. You may also include additional comments for the author, including concerns about dual publication, research ethics, or publication ethics. (Please upload your review as an attachment if it exceeds 20,000 characters)

Reviewer #1: The authors propose a model for honey bee colony collapse that originates when foragers infected with Nosema ceranae engage in tropohallactic exchange with uninfected nectar-receivers. Their model is based on a series of parameters (e.g., egg-laying rate, duration of caste-specific roles, forager homing probability) whose input into deterministic and stochastic simulations establishes scenarios under which a colony may fail from Nosema disease. Most parameter values are supported by the literature. The model also considers the role of social segregation and hygienic behavior in creating first and second lines of defense against the spread of N. ceranae from forager to nectar-receiver to nurse bee to brood; this sequence of contact is important to the model. The take home message is that a large colony with a strong nurse bee population can buffer a high level of infection as long as the rate of egg-laying surpasses the rate of hygienic removal of infected individuals. In stochastic simulations, it is not only colony size but the failure of infected foragers to return to the colony coupled to social segregation (i.e., lack of contact of uninfected nectar-receivers with infected foragers) that will increase chances of colony survival.

Of concern is that the model relies heavily on two assumptions that have not received much attention in our understanding of Nosema disease: oral-oral transmission and infection of immature stages. A widely-accepted route of horizontal transmission of Nosema in honey bees is fecal-oral where bees ingest spores from contaminated feces. Moreover, acquisition of spores by this route may not necessarily be restricted to foragers outside the hive, as is assumed by the model. Oral-oral transmission has been tested under laboratory conditions using caged bees (Smith, 2012); however, there has been little additional work in this area. A justification for why oral-oral transmission was the preferred route should be made more apparent.

The second area of concern is the assumption that immatures become infected under natural conditions. A few studies have shown that immatures can become infected under controlled/laboratory conditions. In addition, Urbieta-Margo et al. (2019) demonstrate natural infection, but at very low levels of infection using PCR methods. What remains to be shown is histological evidence of Nosema development within tissues of immatures collected from colonies. Furthermore, if it is accepted that immatures become infected, and that bees performing hygienic behavior remove infected immatures, then wouldn't the sequence of infection also be from immature to adult instead of only adult to immature as per the model? It is common to see pupae that have been “chewed-down” midway through removal by hygienic bees. During this removal, if the immature was infected, then it would seem that adults performing removal may become infected by handling contaminated tissues. Perhaps the model should also be run without the inclusion of infected immatures and hygienic behavior acting on individuals in this stage?

Minor Comments:

Overall, the manuscript is well-written. There are some language and usage issues; therefore, additional editing is recommended.

Line 5-6: Consider that some evidence shows stabilization or increase in honey bee populations worldwide (e.g., data from FAO).

Line 58: APV is meant to be Acute bee paralysis virus (ABPV)?

Line 250: It is not clear but does the model accommodate acceleration of behavioral development and premature death of non-infected bees responding to loss of infected bees?

Reviewer #2: See attached document.

Reviewer #3: Review – PONE-D-20-25709 – A Model of Nosema ceranae Infection in Honeybee Colonies with Social Immunity

Summary of Comments

Main Comments

This is one of many technically good mathematical models attempting to understand the dynamics and infection processes underpinning honey bee colony loss. Whilst the authors’ capturing of interaction structure and social hygienic behaviours is of interest, worthy of pursuit, and will contribute to the field, the current paper sits an awkward place of alleging to be pathogen specific without doing an adequate job of capturing that pathogen’s biology (see some points below). However, the presentation of the manuscript lacks generality or reference to which other pathogens it might represent, and where it sits comparatively with other demographic or epidemiological models. I have given some discussion below as to my objections to some of the core biology underpinning the model, which will need revision.

I do believe this work will be useful, but I recommend the authors commit to either: 1) a Nosema ceranae specific model with much greater exploration of the true biology of the pathogen, including its density-dependant airborne transmission and facultative precocial foraging by bees upon infection; or 2) a much more generic model where they explore parameter space more generally, remove their claims to this being a specific study of the Nosema system, and more generally overview which pathogens exhibit biology close to what they are modelling. I also believe they should better explicitly reference what their model does similarly and differently to other models in this system, some of which they reference well, others poorly, and some are missed.

Introduction

The introduction is long for the paper and will likely require some changes following my recommendations below.

In particular, Lines 1-21: This section reads as extremely generic and, in places, it is incorrect. For example, ‘CCD’ has been well hypothesised as a symptom of elevated forager mortality following honey bee modelling work by Khoury et al 2011, work which the authors do cite later in the introduction but without this clarity. Much of the reference to CCD, which is no longer considered a useful term by honey bee biologists, seems to be done as a generic replacement for colony death (precisely why we now discourage its use). The authors state that worldwide, colonies are declining (Line 5). This is simply incorrect (see UN FAO data showing worldwide increase in honey bee colonies despite declines in parts of NW Europe and North America).

I also believe the authors miss some critical studies honey bee mathematical modelling which their work should be framed against, for example Booton et al 2017 https://www.sciencedirect.com/science/article/abs/pii/S0022519317301212 and Bartlett et al and Bartlett et al 2019 https://besjournals.onlinelibrary.wiley.com/doi/full/10.1111/1365-2664.13461

both of which include aspects of this work which are, to my view, missed. For theory to remain cohesive and integrated within the field of honey bee biology, more explicit reference must be made to similarities and differences between model studies. I would ask the authors to explicitly state in the introduction which parts of other models they capture, which they do not, and what they introduce which is new in the context of honey bee mathematical modelling.

Methods, Results, Discussion

The results and discussion are well presented and seem to my reading correct in their derivations. However, I do not believe the core biological assumptions made can be justified. For example, the authors themselves state that there is no evidence of the removal of Nosema infected brood, but include it regardless. I do not object to the modelling in principle, but the study should not be presented as a study of a specific disease system if it does not reflect the known biology. On this topic, it has been experimentally shown that Nosema infections can occur without trophallaxis (a frequency-dependant infection dynamic) simply via close, non-contact proximity (and that in this regard, it is a density-dependant infection dynamic). Roberts & Hughes 2015 (https://www.cambridge.org/core/journals/parasitology/article/horizontal-transmission-of-a-parasite-is-influenced-by-infected-host-phenotype-and-density/B05BBB47CE18A0A7F8323489F2C322FC) showed this specifically for Nosema ceranae in honeybees. Given this, I do not believe the model suitable for the specific studying of Nosema infections in honey bee colonies. If the authors wish to remain specifically studying Nosema, I believe the model must be revised. If they wish to present this as a generic model including both infected culling and infection purely via direct interaction then they must justify this biologically and remove explicit claims of modelling Nosema. This extends to their citing of Goblirsch et al for the additional mortality at the foraging stage of Nosema infection. Goblirsch et al explicitly state that infection with Nosema induces preferential abandonment of in-colony duties and a precocial foraging, which would have serious implications for their models behaviour and the conclusions to be drawn from it. I realise the authors mention this extension in line 473-475, but I believe the importance of this point to justifies its inclusion in the original model.

I do however believe the model framework in how it addresses hygienic behaviour & social culling, forager/ receiver / onward transmission, and the implications thereof to be strong and worth publishing with modification, although I do also believe that a flexible task allocation and infection-dependant precocial ageing into the forager class should be included in this (see above reference and many others).

I have not given detailed comments beyond this, as I consider these biological problems with the model’s mandate to require enough revision that additional commentary on more detailed aspects of the work to not remain relevant. However, I would be happy grateful to review this manuscript again in more detail if the authors agree with some of these recommendations.

Conclusion

Similarly, I expect part of the conclusion to change if the biological rationale is improved. I also mirror my comments regarding the introduction that the conclusion is very generic in its discussion around compound stressors and alleged ‘CCD’, for example Lines 433-445 have little relevance to the work here as they are currently written. If the authors wish to discuss a variety of pathogens of honeybees, it should be done with regards to their known biology, and whether the interaction structure and social hygienic behaviours they present would behave similarly or differently. It is critical the authors make it clear what parasites they believe these findings to apply to, and which they do not. Note I do not believe this model currently approximates Nosema biology well enough to be stated as a study of Nosema ceranae infection dynamics.

**********

6. PLOS authors have the option to publish the peer review history of their article (what does this mean?). If published, this will include your full peer review and any attached files.

If you choose “no”, your identity will remain anonymous but your review may still be made public.

**Do you want your identity to be public for this peer review?** For information about this choice, including consent withdrawal, please see our Privacy Policy.

Reviewer #1: No

Reviewer #2: No

Reviewer #3: No

[NOTE: If reviewer comments were submitted as an attachment file, they will be attached to this email and accessible via the submission site. Please log into your account, locate the manuscript record, and check for the action link "View Attachments". If this link does not appear, there are no attachment files.]

While revising your submission, please upload your figure files to the Preflight Analysis and Conversion Engine (PACE) digital diagnostic tool, https://pacev2.apexcovantage.com/. PACE helps ensure that figures meet PLOS requirements. To use PACE, you must first register as a user. Registration is free. Then, login and navigate to the UPLOAD tab, where you will find detailed instructions on how to use the tool. If you encounter any issues or have any questions when using PACE, please email PLOS at figures@plos.org. Please note that Supporting Information files do not need this step.

Attachment Submitted filename: PONE_manuscript_082920_report.pdf Click here for additional data file.

Attachment Submitted filename: Review Comment PONE-D-20-25709.pdf Click here for additional data file.

# Author comment: Author response to Decision Letter 0

**DOI:** 10.1371/journal.pone.0247294.r002

18 Jan 2021

We have tried our best to address all concerns and suggestions raised by the editor and reviewers by providing point-by-point responses to the comments. Please see our 'Response to Reviewers' file.

Attachment Submitted filename: Response to Reviewers.pdf Click here for additional data file.

# Peer review: Decision Letter 1

**DOI:** 10.1371/journal.pone.0247294.r003

**Authors:** Adam G Dolezal

5 Feb 2021

A Model of Infection in Honeybee Colonies with Social Immunity

PONE-D-20-25709R1

Dear Dr. Laomettachit,

We’re pleased to inform you that your manuscript has been judged scientifically suitable for publication and will be formally accepted for publication once it meets all outstanding technical requirements.

Within one week, you’ll receive an e-mail detailing the required amendments. When these have been addressed, you’ll receive a formal acceptance letter and your manuscript will be scheduled for publication.

An invoice for payment will follow shortly after the formal acceptance. To ensure an efficient process, please log into Editorial Manager at http://www.editorialmanager.com/pone/, click the 'Update My Information' link at the top of the page, and double check that your user information is up-to-date. If you have any billing related questions, please contact our Author Billing department directly at authorbilling@plos.org.

If your institution or institutions have a press office, please notify them about your upcoming paper to help maximize its impact. If they’ll be preparing press materials, please inform our press team as soon as possible -- no later than 48 hours after receiving the formal acceptance. Your manuscript will remain under strict press embargo until 2 pm Eastern Time on the date of publication. For more information, please contact onepress@plos.org.

Kind regards,

Adam G Dolezal

Academic Editor

PLOS ONE

Additional Editor Comments (optional):

Both of the reviewers agree the manuscript is improved and is now acceptable for publication.

On one very small note, I recommend double checking usage of conventions on the use of names of viruses (e.g., first use and subsequent abbreviation use) and other taxa (e.g. Varroa) in writing. (https://wwwnc.cdc.gov/eid/page/scientific-nomenclature or similar website). I think any changes to this that the authors may want to make can be done in the proof stage.

Reviewers' comments:

Reviewer's Responses to Questions

**Comments to the Author**

1. If the authors have adequately addressed your comments raised in a previous round of review and you feel that this manuscript is now acceptable for publication, you may indicate that here to bypass the “Comments to the Author” section, enter your conflict of interest statement in the “Confidential to Editor” section, and submit your "Accept" recommendation.

Reviewer #1: All comments have been addressed

Reviewer #2: All comments have been addressed

**********

2. Is the manuscript technically sound, and do the data support the conclusions?

The manuscript must describe a technically sound piece of scientific research with data that supports the conclusions. Experiments must have been conducted rigorously, with appropriate controls, replication, and sample sizes. The conclusions must be drawn appropriately based on the data presented. 

Reviewer #1: Yes

Reviewer #2: Yes

**********

3. Has the statistical analysis been performed appropriately and rigorously? 

Reviewer #1: Yes

Reviewer #2: N/A

**********

4. Have the authors made all data underlying the findings in their manuscript fully available?

The PLOS Data policy requires authors to make all data underlying the findings described in their manuscript fully available without restriction, with rare exception (please refer to the Data Availability Statement in the manuscript PDF file). The data should be provided as part of the manuscript or its supporting information, or deposited to a public repository. For example, in addition to summary statistics, the data points behind means, medians and variance measures should be available. If there are restrictions on publicly sharing data—e.g. participant privacy or use of data from a third party—those must be specified.

Reviewer #1: Yes

Reviewer #2: Yes

**********

5. Is the manuscript presented in an intelligible fashion and written in standard English?

PLOS ONE does not copyedit accepted manuscripts, so the language in submitted articles must be clear, correct, and unambiguous. Any typographical or grammatical errors should be corrected at revision, so please note any specific errors here.

Reviewer #1: Yes

Reviewer #2: Yes

**********

6. Review Comments to the Author

Please use the space provided to explain your answers to the questions above. You may also include additional comments for the author, including concerns about dual publication, research ethics, or publication ethics. (Please upload your review as an attachment if it exceeds 20,000 characters)

Reviewer #1: (No Response)

Reviewer #2: (No Response)

**********

7. PLOS authors have the option to publish the peer review history of their article (what does this mean?). If published, this will include your full peer review and any attached files.

If you choose “no”, your identity will remain anonymous but your review may still be made public.

**Do you want your identity to be public for this peer review?** For information about this choice, including consent withdrawal, please see our Privacy Policy.

Reviewer #1: No

Reviewer #2: No

# Editor report: Acceptance letter

**DOI:** 10.1371/journal.pone.0247294.r004

**Authors:** Adam G Dolezal

12 Feb 2021

PONE-D-20-25709R1 

A Model of Infection in Honeybee Colonies with Social Immunity 

Dear Dr. Laomettachit:

I'm pleased to inform you that your manuscript has been deemed suitable for publication in PLOS ONE. Congratulations! Your manuscript is now with our production department. 

If your institution or institutions have a press office, please let them know about your upcoming paper now to help maximize its impact. If they'll be preparing press materials, please inform our press team within the next 48 hours. Your manuscript will remain under strict press embargo until 2 pm Eastern Time on the date of publication. For more information please contact onepress@plos.org.

If we can help with anything else, please email us at plosone@plos.org. 

Thank you for submitting your work to PLOS ONE and supporting open access. 

Kind regards, 

PLOS ONE Editorial Office Staff

on behalf of

Dr. Adam G Dolezal 

Academic Editor

PLOS ONE
